# Mapping the Environmental Fitness Landscape of a Synthetic Gene Circuit

**DOI:** 10.1371/journal.pcbi.1002480

**Published:** 2012-04-12

**Authors:** Dmitry Nevozhay, Rhys M. Adams, Elizabeth Van Itallie, Matthew R. Bennett, Gábor Balázsi

**Affiliations:** 1 Department of Systems Biology, The University of Texas MD Anderson Cancer Center, Houston, Texas, United States of America; 2 Department of Biochemistry and Cell Biology and Institute of Biosciences and Bioengineering, Rice University, Houston, Texas, United States of America; ETH Zurich, Switzerland

## Abstract

Gene expression actualizes the organismal phenotypes encoded within the genome in an environment-dependent manner. Among all encoded phenotypes, cell population growth rate (fitness) is perhaps the most important, since it determines how well-adapted a genotype is in various environments. Traditional biological measurement techniques have revealed the connection between the environment and fitness based on the gene expression mean. Yet, recently it became clear that cells with identical genomes exposed to the same environment can differ dramatically from the population average in their gene expression and division rate (individual fitness). For cell populations with bimodal gene expression, this difference is particularly pronounced, and may involve stochastic transitions between two cellular states that form distinct sub-populations. Currently it remains unclear how a cell population's growth rate and its subpopulation fractions emerge from the molecular-level kinetics of gene networks and the division rates of single cells. To address this question we developed and quantitatively characterized an inducible, bistable synthetic gene circuit controlling the expression of a bifunctional antibiotic resistance gene in *Saccharomyces cerevisiae*. Following fitness and fluorescence measurements in two distinct environments (inducer alone and antibiotic alone), we applied a computational approach to predict cell population fitness and subpopulation fractions in the combination of these environments based on stochastic cellular movement in gene expression space and fitness space. We found that knowing the fitness and nongenetic (cellular) memory associated with specific gene expression states were necessary for predicting the overall fitness of cell populations in combined environments. We validated these predictions experimentally and identified environmental conditions that defined a “sweet spot” of drug resistance. These findings may provide a roadmap for connecting the molecular-level kinetics of gene networks to cell population fitness in well-defined environments, and may have important implications for phenotypic variability of drug resistance in natural settings.

## Author Summary

It is common belief that the properties of cells depend on their environment and on the genes they carry. Yet, many cases exist where individual cells in the same environment behave very differently, despite sharing the same genes. This creates a problem when we try to explain the behavior of a cell population based on the genes these cells carry. For example, it is difficult to predict how fast the overall number of cells increases based on the genes they all carry if some cells divide much faster than others. We addressed this problem using a synthetic gene circuit that could randomly allocate cells into drug resistant and drug sensitive states. We could control the fractions of cells and the time they resided in these states by adding an inducer to the growth solution. After measuring how fast cells transitioned between these two states, and how fast they grew in inducer and drug alone, we predicted computationally how fast they should grow when both inducer and drug are present. We validated experimentally these predictions and found a “sweet spot” of drug resistance where cells grew fastest in the presence of drugs.

## Introduction

**Figure 1:**
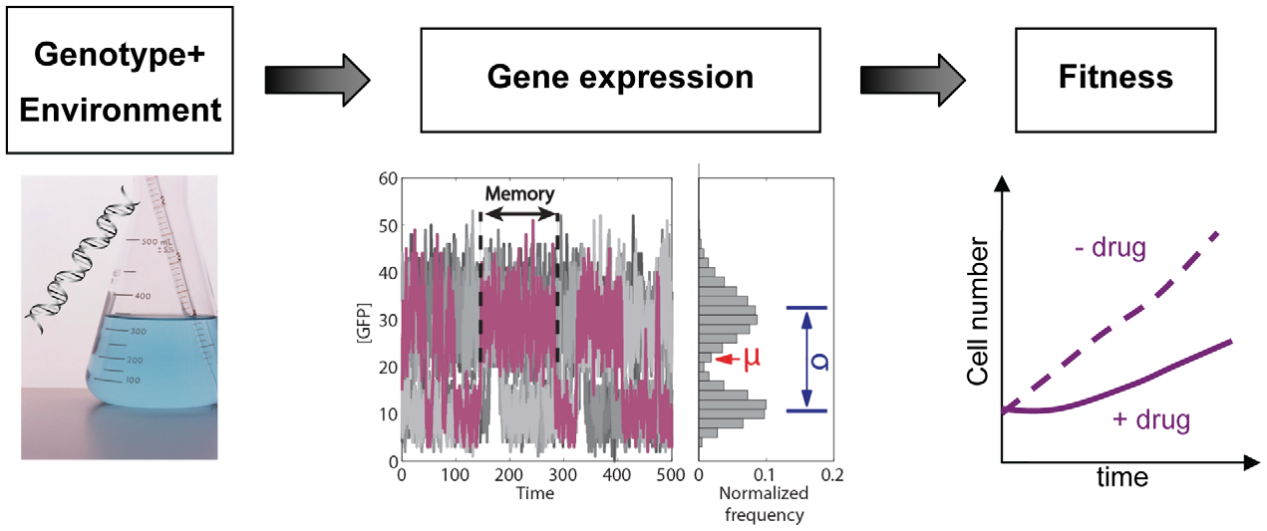
Gene expression connects genotype to phenotype. Gene expression (middle panel) is a complex process that bridges the genotype (left panel) and fitness (right panel). Stochastic gene expression at any instant of time can be described by a distribution (gray bars). The distribution can be further characterized by its mean (red arrow; the arithmetic average taken over all cells), standard deviation (blue arrows; quantifies deviations from the mean), and possibly other moment-related metrics. Moreover, gene expression also has temporal aspects, which can be characterized by nongenetic (cellular) memory (horizontal black arrows; the average time for which cells maintain a specific expression state in a constant environment). Ten time course simulations of stochastic gene expression in bistable cell lineages are shown as illustration (gray traces).

Gene expression is the biological process that converts the cell's genotype into phenotype (Fig. 1) in an environment-dependent manner [1]. From an evolutionary standpoint, *fitness* (cell population growth rate) may be the most important phenotype encoded, since it defines the competitive ability of a genotype in specific environments. The critical role of gene expression as a determinant of fitness in various environments was confirmed by a variety of gene expression measurement techniques, including beta-galactosidase assays [2]–[4] and microarrays [5]–[7]. Such techniques typically rely on pooling millions of cells, and therefore can only measure the average gene expression of a given sample. Consequently, many studies in the past have tacitly used the population *average* of gene expression as a proxy for “gene expression”. However this may be problematic due to the lack of information of how the gene expression of individual cells differs from the population average [8].

Accumulating evidence indicates that cells with identical genomes exposed to the same environment can differ dramatically in their gene expression and phenotype [9]–[13]. Protein levels can vary from cell to cell due to pre-existing differences in cellular characteristics and microenvironments, or simply due to the stochastic nature of intracellular biochemical events [14]–[19]. The ubiquity of non-genetic heterogeneity in clonal cell populations implies that gene expression is a complex stochastic process incompletely described by the mean alone. Rather, gene expression in a cell population is more accurately represented by a distribution, and requires further statistical descriptors (such as the standard deviation, variance, or coefficient of variation) to quantify the amplitude of deviations from the population mean (Fig. 1). Recent experiments showed that cell populations with identical gene expression means, but different gene expression variances survive differently in stress [20]–[22]. Yet, an important aspect that received less attention is that gene expression variation (noise) can also correspond to variable cell division rates. Consequently, the average *fitness* (cell population growth rate) may not reflect the division rates of individual cells. It is not entirely clear how to link the molecular-level kinetics of gene networks to the variable division rates of single cells and to cell population growth rate in specific environments.

**Figure 2:**
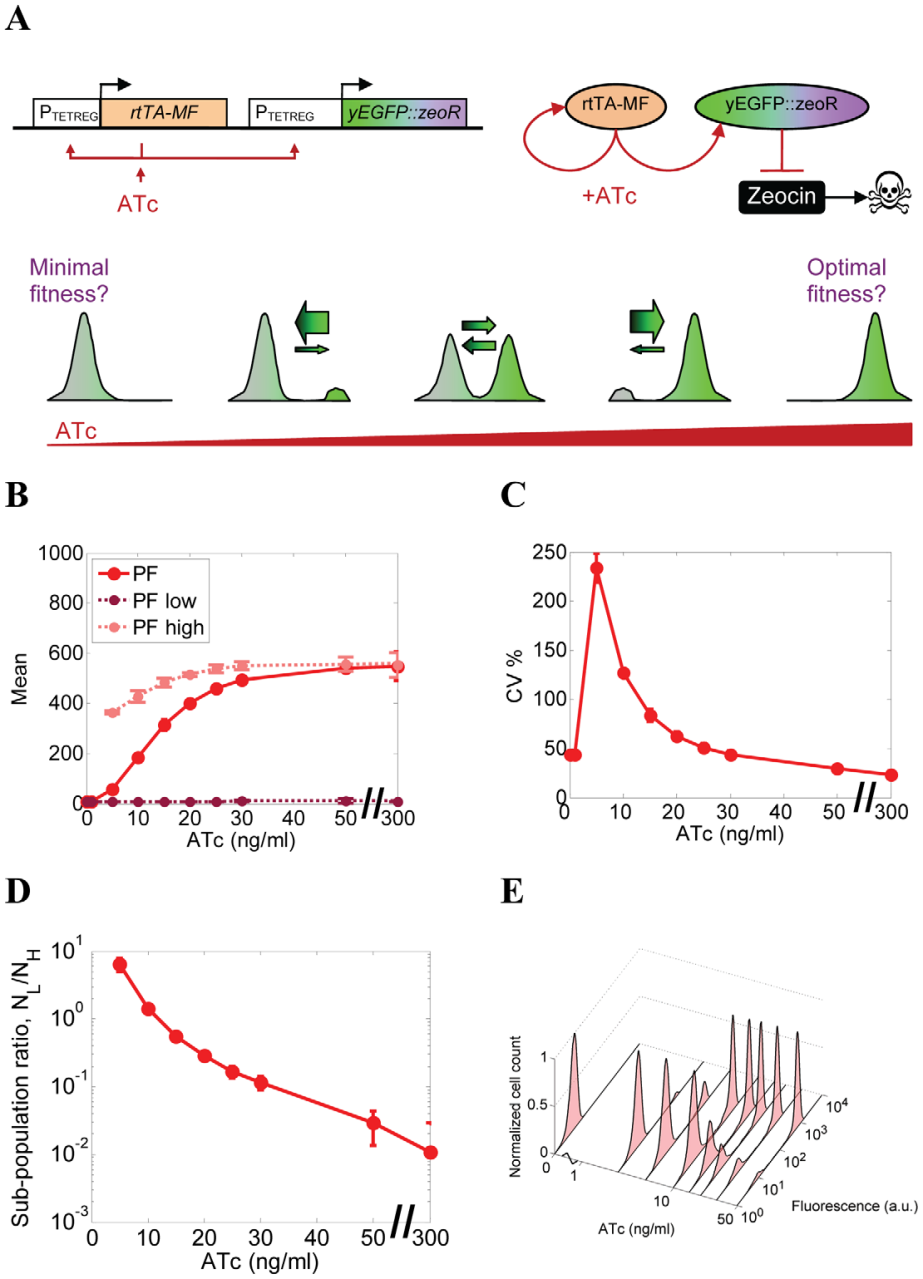
Characterization of gene expression in cells bearing PF circuit. (A) The positive feedback (PF) synthetic gene circuit and its functional schematic, consisting of the *rtTA-MF* transactivator that activates itself and the *yEGFP::zeoR* reporter when bound by the ATc inducer. Idealized plots of expected outputs illustrates that tuning the inducer concentration upward is expected to result in progressively higher fractions of *yEGFP::zeoR*-expressing ON cells (green peaks), which should correspond to increasing fitness during drug treatment. Another naïve expectation is that increasing fractions of ON cells correspond to higher cellular memory of ON cells relative to the OFF cells, such that equal memories (or switching rates, indicated by the arrows) correspond to equal fractions of ON and OFF cells. (B) Dose-response of the population average (mean) of yEGFP::ZeoR expression controlled by the PF gene circuit. Mean fluorescence values are shown for overall cell populations, as well as for low and high expressor subpopulations separately (dark and light red markers) where applicable, based on a custom bimodality detection algorithm (see Section 2 in Text S1). (C) Dose-response of the overall coefficient of variation (CV) of yEGFP::ZeoR expression for the PF gene circuit. (D) Dose-response of the subpopulation ratio *R*, defined as the number of low *yEGFP::ZeoR*-expressing cells divided by the number of high *yEGFP::ZeoR*-expressing cells. (E) Experimental fluorescence histograms of *yEGFP::zeoR* for the PF strains at increasing ATc concentrations (0–50 ng/mL).

Deviations of gene expression from the population average are particularly evident when gene expression is bimodal and cells belong to distinct “ON” and “OFF” subpopulations of drastically different protein content. Importantly, cells may switch randomly between such gene expression states, only transiently residing in a given subpopulation. This average time for which an individual cell remains in a given gene expression state defines *cellular memory* [23] (Fig. 1). Bistability and stochastic switching have received substantial attention over the last decade due to growing evidence for their involvement in cellular decision-making and differentiation [12], [24]–[27]. This motivated recent efforts in synthetic biology to design positive feedback gene circuits that function as “cellular memory modules”, maintaining a target gene over multiple cell divisions in one of two or more distinct expression states [28]–[33]. However, tuning cellular memory appears inherently coupled to changes in subpopulation balance: gene expression states become more populated as their stability increases (Fig. 2A). Could it be possible for an unpopulated gene expression state to be nevertheless much more stable than a highly populated state?

Besides the possible practical applications of synthetic memory modules, they can also be used to address fundamental questions on bet hedging, a frequently revisited topic in economics, ecology and evolutionary biology [34]–[36]. The essence of bet-hedging is to preemptively assign parts of a population to diverse survival abilities in expectance of unpredictable external changes. Individual cells may stochastically transition in and out of these protected and usually costly phenotypic states. For example, specific switching rates between a stress-resistance gene's “ON” and “OFF” expression states can optimize population survival in recurrent stress [37]–[40]. Moreover, high variance in the duration of competence episodes optimizes DNA uptake in *Bacillus subtilis*, suggesting that the *variability* of temporal gene expression characteristics can have functional relevance [41]. Yet, most studies so far have assumed that phenotype switching rates can change without an internal fitness cost, and focused on a constant, single fitness level associated with each phenotypic variant in each environment. What happens if altering the time scales of phenotypic fluctuations involves a fitness cost? And how do such convoluted changes of fitness and memory affect optimal switching rates and the tolerance to increasing levels of sustained stress? These important questions remain to be examined experimentally.

To address these questions *using a specific synthetic system*, we developed and quantitatively characterized a chromosomally integrated, inducible synthetic gene circuit that enforced bimodal distribution of a bifunctional protein (yEGFP::ZeoR) in *Saccharomyces cerevisiae* [42]. This bifunctional protein served as a fluorescent reporter while also protecting yeast cells from the antibiotic Zeocin, enabling us to directly relate gene expression variability to the corresponding fitness variations.

Proper understanding of the environment-fitness connection would imply that we can predict the phenotype (fitness) given the genotype and the environment. Therefore, we first characterized cell population fitness in two specific sets of environmental conditions (i.e., antibiotic alone and inducer alone), and aimed to make experimentally testable fitness predictions in new environments defined as combinations of the same inducer and antibiotic. We found that the nongenetic (cellular) memory of *yEGFP::zeoR* expression *and* individual cell division rates at specific gene expression states were necessary to predict overall cell population fitness with a high degree of precision. Based on these considerations, we identified a specific inducer-antibiotic combination that defined a “sweet spot” where cells maximized their drug resistance due to cellular memory. This “sweet spot” appears for different reasons than the optimum predicted by recent bet-hedging models [37]–[40], and does not co-locate with it. The reason for this disagreement is the fitness cost associated with increasing the memory of the drug resistant state in our system. This effect was not considered in recent bet hedging models [37]–[40], but is likely wide-spread in nature, due to the metabolic cost or toxicity of various survival mechanisms, including drug resistance protein expression.

## Results

### A synthetic gene circuit confers bistable expression to a drug resistance gene

We constructed a chromosomally integrated synthetic gene circuit in yeast, which controlled the expression of a bifunctional fluorescent reporter and antibiotic resistance gene, *yEGFP::zeoR* [42]. This “positive feedback” (PF) gene circuit consisted of a modified version (rtTA-MF) of the rtTA activator [43] that, upon binding to the inducer ATc, activated its own expression as well as the expression of *yEGFP::zeoR* from synthetic P_TETREG_ promoters [29] (Fig. 2A). The degree of rtTA activation was adjusted through varying the concentration of the inducer anhydrotetracycline (ATc) in the growth medium, which diffuses through the cell membrane, associates with rtTA and promotes its binding to *tetO2* sites near the TATA box of the target promoter.

We used flow cytometry to collect steady-state gene expression measurements at increasing inducer (ATc) concentrations from an isogenic yeast cell population carrying the PF gene circuit. We repeatedly resuspended the cells into identical medium every 12 hours until the gene expression histograms stabilized. We established dose-response curves (Fig. 2B, C) based on these steady-state measurements, by calculating the *yEGFP::zeoR* reporter expression mean and CV (coefficient of variation = standard deviation divided by the mean; see the Materials & Methods).

As observed previously for similar constructs [29], yeast cell populations carrying the PF gene circuit (called “PF cells” hereafter) had unimodal gene expression at very low induction, and bimodal expression above a low but nonzero ATc threshold (Fig. 2B, D, E and Section 2 in Text S1). The proportion of cells in the high *yEGFP::zeoR* expression state increased with the inducer concentration at the expense of cells with low *yEGFP::zeoR* expression. We quantified this by the subpopulation ratio *R* (Fig. 2D), defined as the number of cells with high *yEGFP::zeoR* expression divided by the number of cells with low *yEGFP::zeoR* expression. The pronounced gene expression bimodality indicated that isogenic PF cells in the same environment separate into high- and low *yEGFP::zeoR* expressors, causing the rise in CV at intermediate ATc concentrations (Fig. 2C, E). This is due to the bistability that arises from combining a nonlinear promoter response with explicit or implicit, growth rate-mediated [44], [45] positive feedback regulation. Based on our earlier insights on related gene expression constructs [42], [46] involving the repressor from which rtTA was derived [43], we developed a mathematical model that agreed with earlier models [29] and the current observation that the PF construct became bistable once the inducer concentration exceeded a threshold value (≈1 ng/ml). These theoretical results (see Section 3 in Text S1) were consistent with the sharply distinct peaks of *yEGFP::zeoR* expression observed experimentally beyond this threshold (Fig. 2B, E).

In biological terms, the bistability of the PF gene circuit implies that PF cells can undergo random phenotypic switching between high and low *yEGFP::zeoR* expression states due to noisy intracellular dynamics. This situation can be depicted as random movement in some potential, which is analogous to Waddington's landscape [47]. Cells will repeatedly dedifferentiate and redifferentiate into these distinct gene expression states as they are forced to explore the landscape under the influence of gene expression noise. Importantly, since the *yEGFP::zeoR* gene product confers resistance to Zeocin, the cells also assume two distinct phenotypes: they can be drug sensitive, low *yEGFP::zeoR* expressors or drug-resistant, high *yEGFP::zeoR* expressors. This leads to the question: how can we utilize this information on the *yEGFP::zeoR* expression pattern, and what else is needed to predict cell population growth rate in well-defined sets of environments defined by various inducer and antibiotic concentrations? The rest of this paper seeks to answer this question.

### Two different types of fitness

Fitness is a central and often controversial concept in evolutionary theory that quantifies the contribution of a given genotype to the next generation [48]–[50]. For historical and practical reasons, there are many different definitions of fitness. For example, models and experiments focusing on competition between genotypes have measured fitness relative to a specific genotype [50], [51]. Alternatively, absolute fitness can be defined as the *per capita* rate of increase in absolute population size [48], [50], [52], [53], which is relevant in non-competitive scenarios. In the remainder of this paper we will adhere to the latter definition, considering a single haploid genotype (PF) that we carefully maintain in exponential growth phase while exposing it to various well-defined environments.

The above gene expression measurements imply an important distinction between two types of fitness that we define below with regard to nongenetic (phenotypic) variation. At intermediate inducer concentrations, two different cell types coexist that constantly convert into each other and potentially divide at different rates. Therefore, individual cell division rates may be very different from the overall rate of cell population increase. For example, in the presence of Zeocin, high *yEGFP::zeoR*-expressing cells will divide much faster than their low *yEGFP::zeoR*-expressing peers. To account for this effect, we define *instantaneous or transient cellular fitness* as the typical rate of division for single cells of a given fluorescence, at specific inducer and antibiotic concentrations. On the other hand, any change in cell population size over time results from individual cells dividing while they randomly switch between various gene expression states. The rate of clonal cell population growth will therefore be described by the *overall cell population fitness* [53]. These notions correspond to the individual and absolute fitness, respectively, as defined in [50], except that we emphasize random fitness changes both from cell to cell and over time. The precise relationship between the two types of fitness will be established below. Our goal will be to understand how the genotype and the environment jointly determine the overall cell population fitness.

### Defining the environmental fitness landscape of uninduced PF cells at various levels of Zeocin

Understanding the environment-fitness connection for drug resistance implies that we can predict the overall cell population fitness given the genotype and the environment. Therefore, we set out to predict computationally the fitness of PF cell populations in arbitrary inducer and drug concentrations based on two pieces of information: (i) the fitness of uninduced cells (drug-sensitive state, ATc = 0) in various Zeocin concentrations; and (ii) the probability of cells residing in Zeocin-resistant and Zeocin-sensitive states at various inducer concentrations, in the absence of drug (Zeocin = 0). Since we already knew the latter from the dose-response (Fig. 2D), we measured experimentally the fitness *g_E_* of uninduced (ATc = 0) PF cell populations at various Zeocin concentrations over several days, using the formula
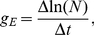
(1)where *N* is the number of cells and Δ*t* is the time between two subsequent cell count measurements. We resuspended the cells every 12 hours into fresh Zeocin-containing medium to maintain them in exponential growth, and applied a linear fit to obtain robust estimates of *g_E_* (see Section 7 in Text S1).

**Figure 3:**
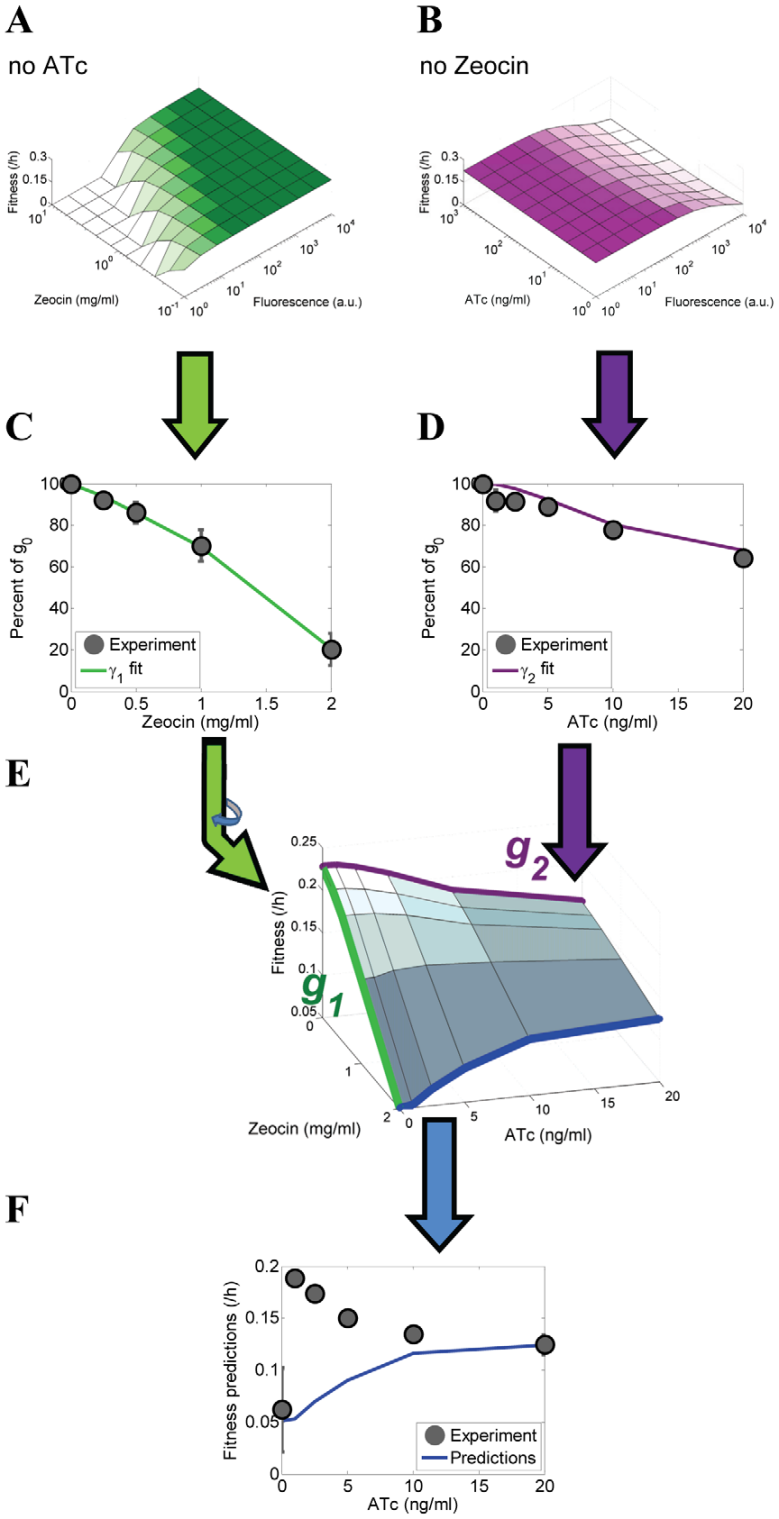
Experimentally measured and predicted fitnesses of PF cells assuming fast switching at various combinations of ATc and Zeocin. (A) Instantaneous fitness reduction *γ*_1_ as a function of varying Zeocin concentrations and fluorescence at 0 ng/ml ATc. The antibiotic prolongs the cell cycle, thereby reducing the instantaneous fitness (which is the typical division rate of cells with a certain level of yEGFP::zeoR expression). (B) Instantaneous fitness reduction *γ*_2_ as a function of varying inducer (ATc) concentrations and fluorescence (used as a proxy for rtTA concentrations) in the absence of antibiotic (0 mg/ml Zeocin). The cell cycle slows down due to the toxic effects of activated rtTA molecules, causing an instantaneous fitness cost as noise forces the cells to explore the potential landscape. (C) Overall population-level fitness reduction as a function of increasing Zeocin concentrations at 0 ng/ml ATc, reflecting the toxic effect of the antibiotic. (D) Overall population-level fitness reduction as a function of increasing ATc concentrations at 0 mg/ml Zeocin due to various costs related to inducer-bound rtTA. (E) Predicted overall population-level fitness landscape for PF cells as a function of various extracellular ATc and Zeocin concentrations. The fitness landscape is calculated by averaging the product of instantaneous fitness reductions *γ*_1_(*F*,*Z*) and *γ*_2_(*F*,*C*), shown in panels (A) and (B) weighted by the fluorescence distributions shown in Fig. 2E. (F) Measured and predicted overall fitness of PF cell populations at different concentrations of ATc and Zeocin = 2 mg/ml. Predictions based on the fitness landscape (blue) explained <0.1% of overall cell population fitness.

Using simple biochemical considerations (Materials and Methods), we defined the instantaneous fitness of uninduced PF cells semi-phenomenologically by the marginal fitness reduction *γ*_1_ (shown in Fig. 3A). The value *γ*_1_(*Z*) refers to various extracellular Zeocin concentrations (*Z*) and is normalized by fitness in the absence of Zeocin. We then calculated the *overall cell population fitness* by averaging the instantaneous fitness reduction over all fluorescence values, weighted by the fluorescence distribution *p*(*F*) (Fig. 3A, C):
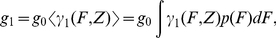
(2)where *g*_0_ is the maximal fitness (at Zeocin = 0 and ATc = 0) and *p*(*F*) is the fluorescence distribution determined from flow cytometry measurements (Fig. 2E). A similar calculation based on arithmetic averaging was used recently by another group [54]. This is a common way of defining overall fitness in genetically mixed cell populations [55], although we average here over phenotypic (rather than genetic) variants. We obtained the parameters determining internal Zeocin concentrations by fitting the function *g*_1_(*F*,*Z*) to the overall cell population fitness values *g_E_* measured in increasing Zeocin concentrations at ATc = 0 ng/ml (Fig. 3C). Parameter values are listed in the Materials and Methods. Further details can be found in Section 5, .

In conclusion, measuring the fitness of uninduced PF cell populations at various levels of Zeocin revealed a gradually decreasing fitness landscape (Fig. 3C) that could be traced to typical cell division rates of individual cells by simple biochemical considerations.

### Defining the environmental fitness landscape of PF cells in the absence of antibiotic

Since our goal was to predict overall cell population fitness at arbitrary inducer (ATc) and antibiotic (Zeocin) combinations, we had to test whether varying the inducer concentration had any fitness impact itself. Therefore, we followed the approach described above to experimentally measure the overall PF cell population fitness at various ATc concentrations, in the absence of antibiotic. We observed a decline in fitness as the fraction of high *yEGFP::zeoR* expressors became larger at increasing ATc concentrations (Fig. 3B). This decline in fitness may be attributed to a combination of costs related to transcription, translation [56], [57] and rtTA toxicity or “squelching” described previously [58]. The rtTA-specific squelching toxicity arises from the VP16 activation domain of the activator, which may form non-specific transcriptional complexes with general transcription factors, sequestering them from vital cellular processes. Since all of the above processes harm cell fitness, there is an overall cost associated with processes related to active (ATc-bound) rtTA. We characterized the typical rate of division for cells of a given fluorescence at a specific inducer concentration by a semi-phenomenological marginal fitness reduction *γ*_2_ (Figs. 3B, D) as we did for Zeocin alone. Parameter values are listed in the Materials and Methods and further details are provided in Section 5, Text S1.

In summary, ATc-bound activator toxicity implies that defense from Zeocin is costly. The more populated the protected, high yEGFP::zeoR expressing state, the higher the active rtTA expression, and the slower the division rate of individual cells and of the entire cell population.

### Fitness landscape predictions assuming low memory are not confirmed experimentally

To predict cell population fitness in various combinations of the inducer and the antibiotic, we assumed that the joint effects of these molecules followed Bliss independence [59]. This implied that the combined action of these two molecules could be calculated by multiplying their individual effects. This assumption was reasonable, considering the different mechanisms involved in the toxicities of Zeocin and active rtTA. Thus, we calculated the instantaneous fitness reduction *γ*(*F*,*C,Z*) at arbitrary levels of ATc and Zeocin by simply multiplying the previously defined marginal fitness components *γ*_1_(*F*,*Z*) and *γ*_2_(*F*,*C*):
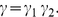
(3)Relaxing the assumption of Bliss independence, and introducing various forms of dependence between Zeocin and ATc-bound activator toxicity did not affect our conclusions (see Section 5 and Fig. S6 in Text S1).

First, we calculated the overall cell population fitness *g_T_* assuming cellular memory was much lower than typical cell division times. Thus, we obtained *g_T_* by averaging the instantaneous fitness reduction, *γ* = *γ*_1_ *γ*_2_, weighting by the fluorescence distribution *p*(*F*) measured in the absence of Zeocin (Fig. 3D) as in equation [2] above. This calculation assumes very fast transitions across the gene expression distribution such that the probability distribution *p*(*F*) remains unchanged after the onset of selection (Zeocin treatment). Based on these assumptions, we predicted the overall cell population fitness *g_T_* for PF cells at different levels of induction (ATc ranging from 0 to 20 ng/ml) with Zeocin concentration set to 2 mg/ml. The predictions showed a mild, gradual rise in fitness as ATc increased (Fig. 3E).

To experimentally test these predictions, we induced PF cells over the range of 0 to 20 ng/ml ATc. After the *yEGFP::zeoR* distributions became stable (did not change from day to day), we exposed these populations to 2 mg/ml Zeocin, and measured the cell density every 12 hours over several days as described above. Fitness was then estimated over the last 6 time points to minimize the transient effects immediately after exposure to Zeocin (see the Materials & Methods and Table S3 in Text S1). We used the R^2^ metric to determine how much of the PF cell population fitness was explained by the computational model. The percentage of the data explained by the fitness landscape was very low, with an R^2^ measured at <0.001, consistent with the disagreement between the predicted and measured overall cell population fitness at low inducer concentrations (ATc<10 ng/ml, Fig. 3F).

### Estimates of cellular memory based on cellular current

Seeking to reconcile the predicted and measured cell population fitness values, we asked whether the fast inter-conversion between various fitness levels used in those calculations was realistic. If not, then slow cellular phenotype switching (cellular memory) may enable individual cells to reside for prolonged times in advantageous regions of the fitness landscape, thus improving overall population fitness [60]. Such an effect was suggested theoretically [37]–[39] and demonstrated experimentally [40] in fluctuating environments, but not in sustained stress.

**Figure 4:**
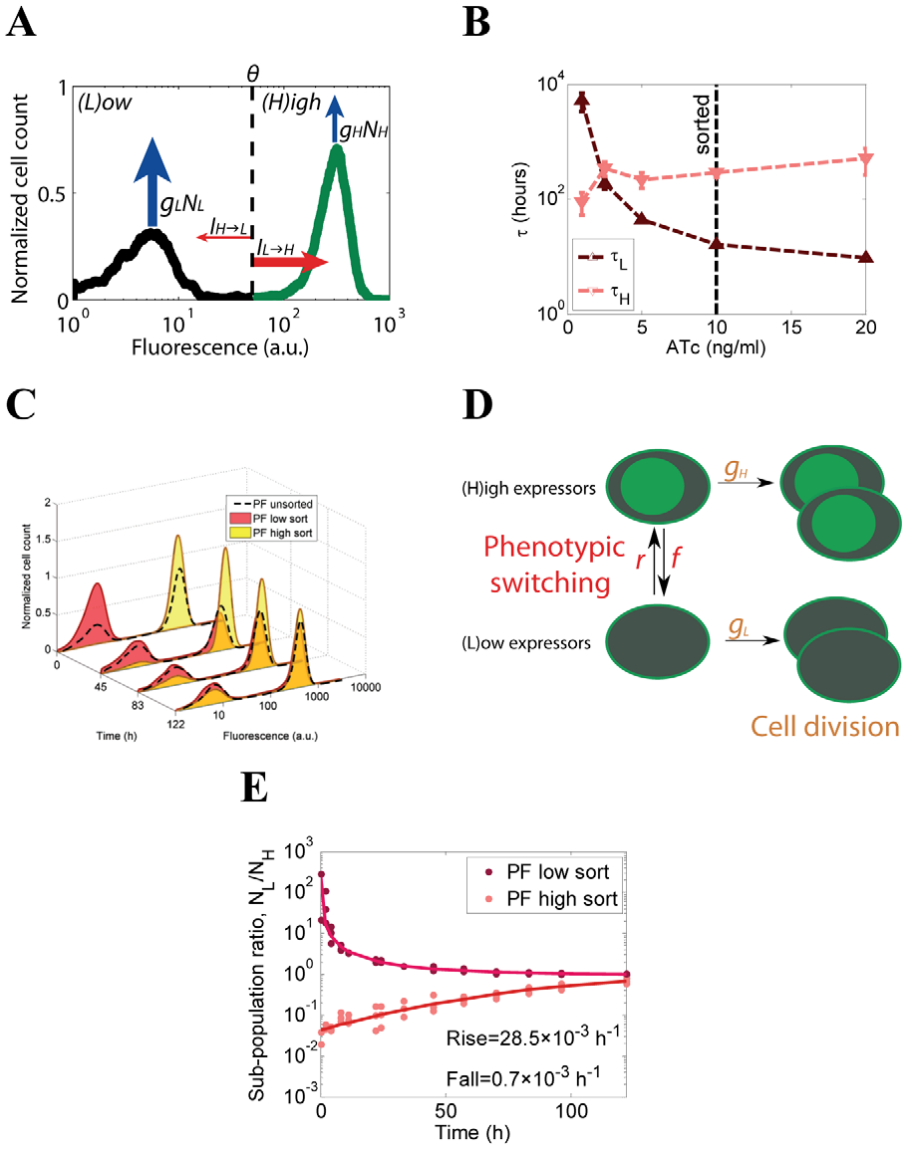
Cellular memory estimation and measurement based on the cellular current. (A) Cellular currents characterize the constant upward and downward movement of cells within stationary distributions under the influence of noise. Cells were first partitioned into 2 gene expression states, of (*L*)ow and (*H*)igh *yEGFP:zeoR* expression, depending on how their fluorescence compared to a preset threshold *θ*. The upward cellular current (*I_L→H_*) was defined as the fraction of cells leaving the *L* gene expression state per unit time, while the downward cellular current *I_H→L_* described movement in the opposite direction. For stationary distributions where fitness is unaffected by particular expression state, the upward and downward cellular currents must cancel each other. On the other hand, unequal high and low expressor fitness values (*g_L_*≠*g_H_*) imply unequal cellular currents (*I_L_*≠*I_H_*), even in stationary distributions. (B) Cellular memories estimated from the gene expression distributions in Fig. 2E using the cellular current model, incorporating instantaneous cellular fitness differences. For example, cellular memory of the *H* state is the average time it takes for a *H* cell to cross the preset threshold downward. (C) Histograms of cells sorted into high and low *yEGFP::zeoR* expression states and maintained in 10 ng/ml ATc-containing medium over several days. Cell sorting occurred at time 0 after fluorescence distributions stabilized (did not change between subsequent measurements). Experimental fluorescence histograms of high-sorted and low-sorted cells relaxing back to the stationary distribution are shown for 122 hours. (D) Schematic of the two state population-dynamics model used to estimate switching rates. (E) Low to high expressor subpopulation ratio (*R* = *N_L_*/*N_H_*) after sorting PF cells into predominantly high and low expressor subpopulations. Experimental time course data (dots) of low-sorted and high-sorted subpopulations and the corresponding fits (lines) based on the two-state population dynamics model illustrate their relaxation back to their original bimodal distributions. The “rising” and “falling” rates were estimated from both low-sorted and high-sorted cell populations, revealing a higher gene expression memory for PF high expressors as compared to low expressors.

We developed a novel computational method related to cell population balance modeling [61] to determine the cellular memory based on a stationary distribution of expression for proteins with long half life, such as yEGFP::ZeoR (see the Section 6 in Text S1). To do this, we defined the *directional cellular current* as the number of cells crossing a specific fluorescence threshold in a given direction (up or down) per unit time. We designated cells into two phenotypes, the (*L*)ow and (*H*)igh *yEGFP::zeoR* expression states, separated by an arbitrary yEGFP::ZeoR concentration threshold, *θ* (Fig. 4A). The *H* and *L* states do not need to imply bistability and are quite general: they simply indicate that cells express *yEGFP::zeoR* below or above a threshold. Importantly, the yEGFP::ZeoR protein is highly stable (data not shown) and is mainly lost through dilution of cellular contents by cell growth [62]. Therefore, the downward current *I_H→L_* of cells escaping from the *H* to *L* state across the threshold mainly depends on the rate at which cell growth dilutes out the protein yEGFP::ZeoR. Thus *I_H→L_* is proportional to: (i) the density of cells ∂*N*(*F*)/∂*F* expressing *yEGFP::zeoR* immediately above the threshold *θ*; and (ii) the rate of protein dilution by cell growth, *θg*_2_(*θ*,*C*), calculated at the threshold *θ*:
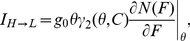
(4)assuming that protein dilution occurred due to cell growth at the rate *g_0_γ*_2_, from equation [3].

Next, we calculated how long it would take on average for an arbitrary cell with high *yEGFP::zeoR* expression to cross the threshold downward. If the total number of cells in the (*H*)igh state is *N_H_*, the average waiting time *τ_H_* spent in the state *H* is given by [63], [64]

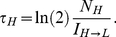
(5)This is equivalent to asking: how long would it take for an arbitrary cell to leave the *H* state, if cells exit at rate *I_H→L_*? The time spent in state *H* is therefore the cellular memory of the high *yEGFP::zeoR* expression state. These calculations provide a simple way to estimate the memory of any gene expression state defined by an arbitrary threshold within any stationary distribution. We also applied other approaches, including escape rate theory [23], [65], [66] to estimate cellular memory and obtained consistent, but less accurate results (see Section 4 in Text S1). Our semi-phenomenological approach based on cellular current may be more attractive because, unlike escape rate theory, it does not require an underlying mathematical model and a set of parameters.

Normally, in the absence of instantaneous fitness differences, when stationary distributions do not change in time, the downward and upward cellular currents across any threshold must be in balance, and as a result the net current is 0: *I_L→H_* = *I_H→L_*. However, when instantaneous fitness changes with the gene expression level, the cellular currents must compensate for these fitness differences (see the Materials and Methods). As a result, cells of higher individual fitness will constantly migrate towards regions of lower fitness in the gene expression space. This creates a nonzero net cellular current that moves cells upward throughout the gene expression distributions in Fig. 2E. The “power sources” for this current are fitness differences across these distributions (Fig. 3A).

We estimated cellular memories at various ATc concentrations by applying the above formula to the experimental yEGFP::ZeoR expression distributions (Fig. 2E) and cell population fitness values (Fig. 4B). We found that soon after the ATc concentration crossed the bistability threshold and some PF cells started to differentiate into a drug-tolerant high expressor subpopulation, these cells gained increasingly higher memory, while the memory of low expressors showed a monotone decrease (Fig. 4B). The decreasing and increasing trends in Fig. 4B indicate that ATc can be used to tune the memory of the high and low expression states (or equivalently, the rates of stochastic transitions between these expression states). These trends mirror the inducer-dependent changes in subpopulation fractions (Fig. 2D), indicating that changes in memory and subpopulation fractions are coupled in the absence of Zeocin, as illustrated in Fig. 2A. In particular, at 10 ng/ml ATc, the memory of high yEGFP::ZeoR was >283 hours, an order of magnitude higher than the memory of low expressing cells (∼16 hours), as indicated by the vertical dashed line in Fig. 4B. Importantly, we had to smooth the experimental fluorescence distributions to deal with empty bins (instances of *N*(*θ*) = 0). As a result, the memory *τ_H_* obtained for high expressors should be considered a conservative lower estimate.

### Experimental confirmation of cellular memory estimates in PF cells

Based on the cellular current, we predicted that the memories of high and low *yEGFP::zeoR* expression at [ATc] = 10 ng/ml should be greater than 283 hours and 16 hours, respectively, with more than an order of magnitude difference between the two. To validate these memory predictions, we measured experimentally the nongenetic memory of high and low gene expression states conferred by the PF gene circuit at [ATc] = 10 ng/ml. We selected this intermediate inducer concentration because it gave approximately balanced high and low expressor subpopulations, which facilitated fluorescence-activated cell sorting (FACS). After preinducing the PF cell population with [ATc] = 10 ng/ml, and allowing the fluorescence distribution to stabilize over 3 days, we sorted the high and low expressors apart. Subsequently, we resuspended each of these sorted subpopulations, as well as the unsorted control cells every 12 hours into the same medium as before sorting to determine how fast the yEGFP::ZeoR distributions relaxed to their original stationary distribution via stochastic switching (Fig. 4C).

To quantify the stochastic switching rates between high and low reporter gene expression from this data, we estimated the switching rates of the following two-state population dynamics model that results directly from the cellular current (Fig. 4D) and has been applied successfully by others to estimate cellular memory [23], [37], [40]:
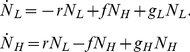
(6)Here, the variables *N_L_* and *N_H_* corresponded to the number of cells in the low (*L*) state that could either “rise” into the high (*H*) state at the rate *r* = *I_L_*/*N_L_*, or divide at the rate *g_L_*, and to the number of cells in the *H* state that could either “fall” into the *L* state at the rate *f* = *I_H_*/*N_H_*, or divide at the rate *g_H_*, respectively. From these equations, we obtained the analytical solution for the subpopulation ratio of low expressors to high expressors over time, *R*(*t*) = *N_L_* (*t*)/*N_H_* (*t*), as a function of *r* and *f* (see the Materials & Methods) (Fig. 4D). Fitting *R*(*t*) to the experimentally measured subpopulation ratios from both sorted and unsorted populations (Fig. 4E), we obtained switching rates of *r*≈28.5±6.6×10^−3^ h^−1^; *f* = 0.7±0.4×10^−3^ h^−1^ for PF cells grown at 10 ng/ml ATc. The obtained PF switching rates correspond to cellular memories of high and low *yEGFP::zeoR* expression of 990 hours and 24 hours, respectively. These values differed by more than an order of magnitude, and compared well with the memory estimates based on the cellular current (Fig. 4B). We made similar predictions and measurements for a few other synthetic gene circuits, confirming the utility of our approach based on cellular current to predict cellular memory (see Fig. S3 in Text S1).

These results indicate that instead of being controlled by comparable rising and falling rates, balanced populations of PF low and high expressors result from low expressor cells preferentially transitioning to high expression, whose lower fitness due to rtTA toxicity prevents them from dominating the population (horizontal arrows on Fig. 4A). Importantly, these effects are completely different from recently noted cases of implicit, growth rate-mediated positive feedback [44], [45]. Rather, a similar case has been described in prokaryotes [9], where different fitnesses compensate for unequal switching rates, re-establishing the subpopulation balance between high and low expressors. Inspired by this classical work on the Lac operon, we further confirmed the strong, but finite non-genetic memory of PF high expressors by purifying single high expressor cells by serial dilution and observing them over several days [9] (see Section 8.2 and Fig. S10 in Text S1). Finally, switching rates estimated from a video recorded in a microfluidic chamber [67] agreed well with the other estimates (see Section 11 in Text S1 and Video S1).

### Overall fitness predictions incorporating cellular memory identify a “sweet spot” of drug resistance

**Figure 5:**
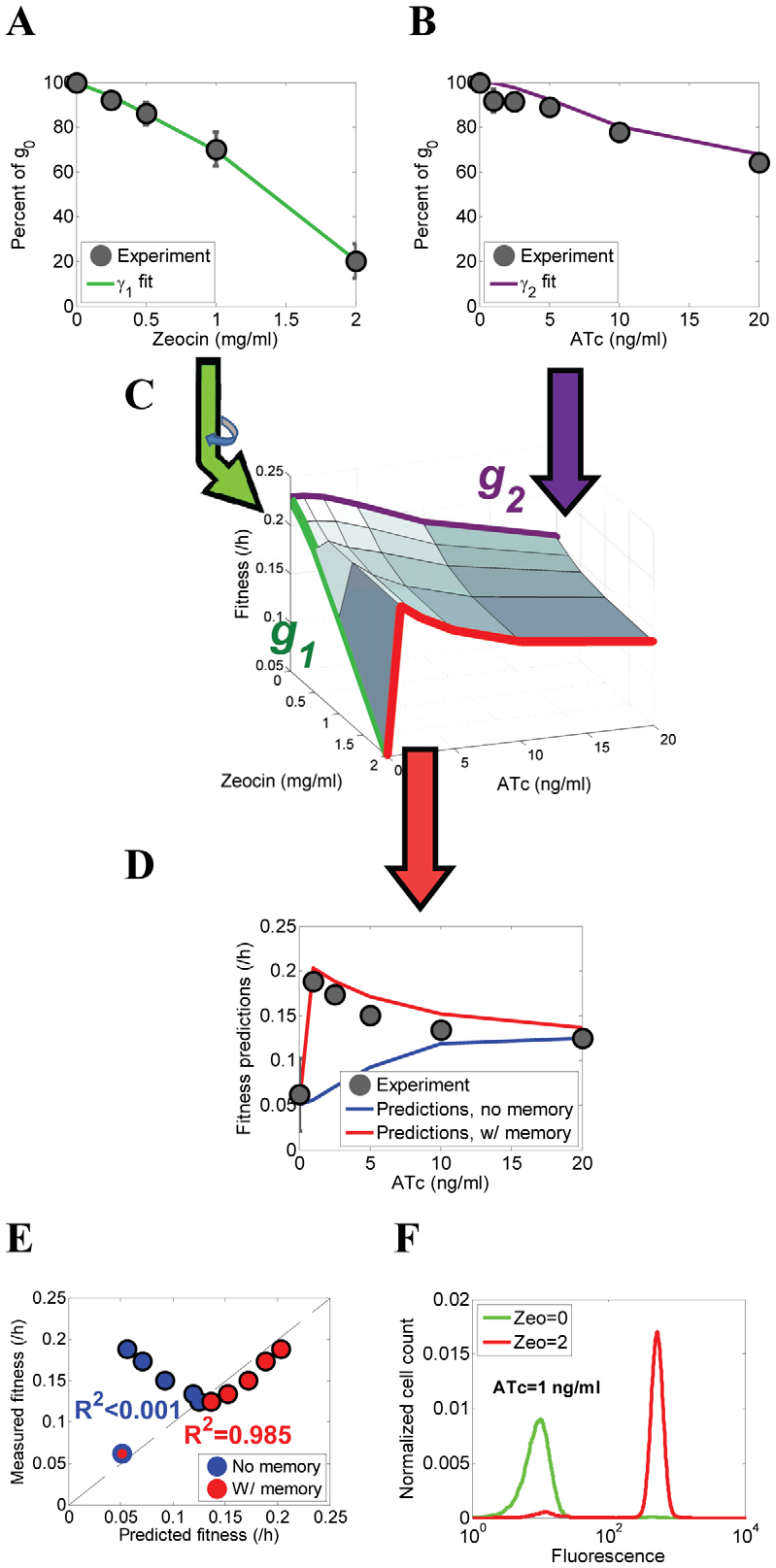
Experimentally measured and predicted fitness of PF cell populations after incorporating cellular memory at various combinations of ATc and Zeocin. (A) Overall population-level fitness reduction as a function of increasing Zeocin concentrations at 0 ng/ml ATc, reflecting the toxic effect of the antibiotic. (B) Overall population-level fitness reduction as a function of increasing ATc concentrations at 0 mg/ml Zeocin due to various costs related to inducer-bound rtTA. (C) Predicted overall population-level fitness landscape for PF cells as a function of extracellular ATc and Zeocin. The fitness landscape is calculated from the 2-state cell model with division rates and switching rates inferred from cellular memory and the marginal fitness functions *γ*_1_(*F*,*Z*) (green edge) and *γ*_2_(*F*,*C*) (purple edge) shown in panels (A) and (B), respectively (D) Experimental measurements (black dots) and computational predictions either incorporating (red line) or omitting (blue line) cellular memory of overall fitness of PF cell populations at different concentrations of ATc and Zeocin = 2 mg/ml. The incorporation of cellular memory improves the agreement between the predictions and experiment. (E) Computational predictions based on the gene expression distributions assuming fast switching (blue circles) explained barely <0.1% of overall cell population fitness, while predictions based on fitness landscape and memory (red circles) explained 98.5% of overall cell population fitness. (F) Experimental yEGFP::ZeoR histograms at the “sweet spot” of drug resistance of 1 ng/ml ATc before selection (Zeocin = 0 mg/ml, green line) and after selection (Zeocin = 2 mg/ml, red line). Note the drastic difference between these two histograms with PF cells changing from almost 100% low expressors to nearly 100% high expressors following selection.

Incorporating the memories obtained from the cellular current [11], [13] (Fig. 4B) as well as fitness values associated with high and low expressors obtained from the instantaneous fitness functions (Fig. 5A, B) , we altered our previous predictions (Fig. 3E) of overall population fitness at a variety of inducer and antibiotic concentrations (Fig. 5C). Using the two-state population dynamics model, which accounts for cellular memory, we determined the overall cell population fitness over long periods of time as the largest eigenvalue of [6] (see the Materials & Methods for the exact expression). Interestingly, the model predicted that population fitness would peak sharply at the lowest level of ATc at which bistability ensued and some cells started switching to the high expression state (Fig. 5D).

The switching rates from the cellular current model predicted that the memory of high expressors would remain well above the cell division rates even when cells were minimally induced. This minimal level of ATc should have minimal cost of rtTA expression (Figs. 3A and 3C), but could still drive high *yEGFP::zeoR* expression in a small fraction of the cells to completely protect them from Zeocin. Importantly, these few “sentinel”, persister-like cells have time to expand into a large population over several days due to the long memory of the protective high *yEGFP::zeoR* expression state at all ATc concentrations, which is necessary to observe this effect.

The inclusion of nongenetic memory into the model dramatically improved the agreement between computational predictions and experimentally measured fitness of PF cells (Fig. 5D, E). Specifically, we found that with no additional information, our model was able to explain 98% of fitness differences simply by incorporating memory (compared to <0.1% without memory). Thus, consistent with the computational predictions, the experiments confirmed that bimodal distributions with fewest high expressors had the greatest relative fitness at ∼1 ng/ml ATc (Fig. 5D), which is where bimodal gene expression first appears. This level of induction defines a “sweet spot”, where the small high expressor subpopulation can reside and expand optimally. Here cells experience sufficient protection from Zeocin at a minimal cost of ATc-bound rtTA, while the strong non-genetic memory of this “protected” gene expression state prevents switching to low expression where cells become vulnerable.

**Figure 6:**
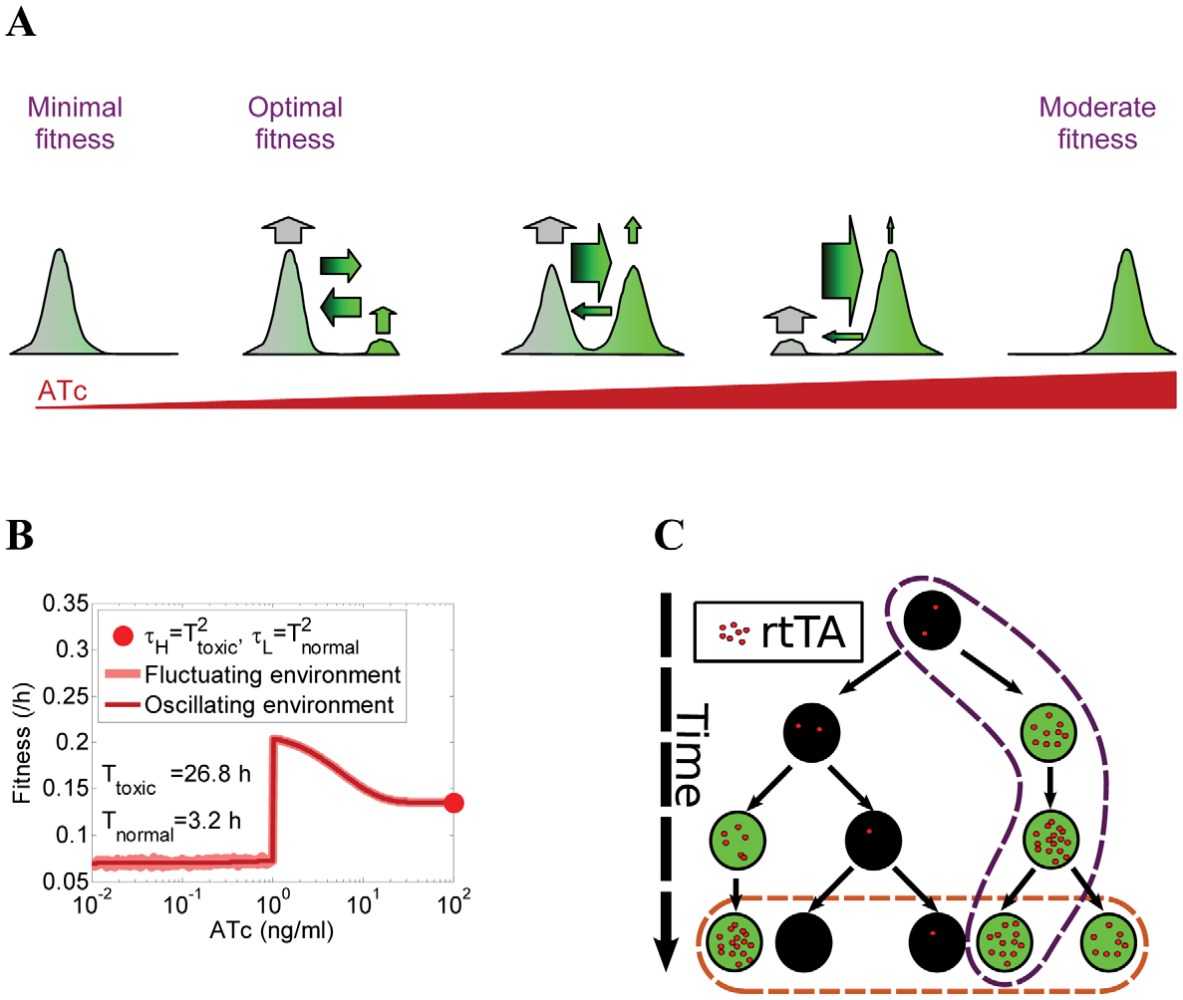
Memory and fitness define the gene expression distribution. (A) The cellular memory and fitness of the cellular states interacted in multiple ways that ultimately defined the overall cell population fitness and the distribution of gene expression. Contrary to the naïve expectations illustrated in Figure 2A, we observed that (i) the fractions of cells in the ON and OFF states did not reflect the switching rates between these states; and (ii) forcing most cells into the protected ON state did not provide optimal fitness during drug treatment. (B) Simulated cell population fitness as a function of ATc when the environment switches either randomly or periodically between 0 mg/ml Zeocin (normal) and 2 mg/ml Zeocin (toxic), with the average duration of the normal and toxic environments indicated in the figure. The red circles indicate the ATc concentration (ATc = 100 ng/ml) necessary for setting the phenotypic switching rates to confer optimal fitness based on the Kussell-Leibler theory. (C) Illustration of how cell linage statistics (purple outline) can be different from population snapshot statistics (orange outline). Cells tend to switch predominantly to the high expression state and rarely switch back. Consequently, individual cell lineages over time tend to spend most of their time in the high expression state. However, cellular fitness in the high expression state is lower. As a result, population snapshot measurements will observe more low expressor cells due to the higher cellular fitness of the low-expressing state.

Importantly, the location of this fitness optimum disagrees with predictions of recent bet-hedging models [37]–[40], which imply that optimal phenotype switching rates should match the rates of environmental fluctuations. Measuring the fitness of PF cells over several days in Zeocin corresponds to very long periods of stress in bet-hedging models (“very long” in terms of cell division times). Thus, according to the previous studies, extended memory of the protective high expressor state should improve survival in long stressful periods (Fig. 6B). However, these earlier conclusions were drawn assuming that nongenetic switching rates could be altered without any fitness cost, and disagree with our observation of a sweet spot at the minimal memory of the high expression state (see the Discussion as well as Section 9 and Fig. S11 in Text S1).

Confirming the crucial role of the minute high expressor subpopulation in conferring protection from Zeocin, the steady state distribution was completely reshaped after selection of nongenetic variants in antibiotic (Fig. 5F). From almost 100% low expressors in the absence of Zeocin, the distribution changed to nearly 100% high expressors, corresponding to a dramatic ∼45 fold increase in the fraction of high expressing cells in Fig. 5F. This implies that subpopulation fractions and cellular memory can be uncoupled for cells carrying gene circuits similar to the one described here, with nongenetic memories longer than their division times. Indeed, the same cellular memory corresponds to very different subpopulation fractions in the absence and presence of drug (Fig. 5F).

We performed parameter scans to determine how various properties inherent to our system and assumptions in our model affect the sharp peak that defines the “sweet spot”. We found that the sharp peak was robust to the inclusion of various interactions between rtTA and Zeocin toxicities (i.e., relaxing the Bliss independence), and remained consistent as long as the cellular memories were less than the overall cell population growth rates. Fitness surfaces generated from other toxicity models based on Loewe additivity, Loewe antagonism, and Loewe synergy all showed a characteristic sweet spot (see Section 5 and Fig. S6, Text S1). However, as the switching rates became comparable to cell division rates, the sharp fitness peak was reduced and became gradually flatter.

## Discussion

Our goal in this paper was to map the environmental fitness landscape, and to identify aspects of stochastic gene expression that are necessary for establishing the environment-fitness connection. This required developing a simple modeling framework to account for the mutual dependency of cell growth and gene expression, so that the behaviors of cells with multiple phenotypes could be better understood and controlled. To allow for predictive quantitative modeling and properly test the modeling predictions, it was necessary to minimize the biological complexity inherent in natural gene networks. Thus, we chose to focus on cell populations carrying an inducible synthetic gene circuit, in the spirit of the booming field of synthetic biology [68]–[72]. The synthetic gene circuit controlled a bifunctional antibiotic resistance protein, which provided protection from Zeocin, and could also be visualized in single cells due to its fluorescence [42].

After determining fitness and subpopulation fractions in two sets of well-defined environments, we used this information to predict fitness and subpopulation fractions in a combination of these environments. This is non-trivial when phenotypic variation is present, because the cell population's average fitness is an aggregate of individual cell fitness values that are transient due to phenotype switching. Thus, to map the environmental fitness landscape, we defined two different types of fitness (transient cellular fitness and overall cell population fitness), which relate to each other as the gene expression in individual cells relates to the population average of gene expression. A similar distinction has existed for genetically diverse populations [53], - but here we defined these types of fitness for genetically homogenous populations of cells that carry the same noisy bistable synthetic gene circuit.

Instantaneous fitness was affected by two different factors, namely the growth-inhibitory effect of Zeocin and rtTA-associated toxicity. We also used the inducer ATc to separately control cellular memory, defined as the average lifetime of a gene expression state (or the inverse of spontaneous switching rates) in a *constant* environment [40], [73], [74]. We found that the instantaneous fitness and cellular memory jointly shape the overall gene expression distribution and determine overall cell population fitness in various environments in a non-trivial manner. Specifically, we argued that the synthetic gene network forces cells to explore an intracellular potential analogous to Waddington's landscape [47] under the influence of noise. While exploring the gene expression landscape, cells are also forced to move on a non-genetic version of Sewall Wright's fitness landscape [75], and face selection through the instantaneous fitness associated with their gene expression. Mapping the environmental fitness landscape requires integrating the movement on these two different landscapes, entirely defined by the cellular memories and instantaneous fitnesses associated with specific cellular states. The interplay between cellular memory and noisy cellular fitness results in distributions and fitness values (Fig. 6A) that disagree with the naïve expectations illustrated in Figure 2A. In summary, a true understanding of the environment-fitness connection can benefit from a Darwinian perspective applied to heritable nongenetic variation [76]. Mapping and computationally predicting the environment-dependent fitness landscape for this synthetic gene circuit might provide a potential roadmap to follow for other synthetic gene circuits and even for natural gene networks, to identify fitness-optimizing conditions relevant for microbial drug resistance, persistence, and virulence.

We strove to develop mathematical models that were as simple as possible, but no simpler, when describing population level gene expression. In isolation, the original goals of these models were to describe mean gene expression levels, stochastic transitions between cell states, rtTA-associated toxicity, and cell growth. We also studied how pairwise interactions between these biological components shape population level fluorescence distributions. Finally, we synthesized these modeling approaches into comprehensive population-level stochastic simulations of gene expression. By modifying previous stochastic models [42], [46] to describe gene activation rather than repression and to account for the cost of inducer-bound activator, cell division, and non-genetic memory we were able to reproduce the overall experimental distributions of PF cells by computer simulations (see Fig. S12 in Text S1). In summary, non-genetic fitness differences between various gene expression states need to be carefully considered when studying instantaneous population-level gene expression measurements based on intracellular biochemical kinetics. Modeling approaches that do not incorporate fitness differences will lead to biased parameter estimates and misinterpretation of intracellular dynamics.

For example, an important implication of our results is that temporal averages over individual cell lineages can be dramatically different from the population means due to slight changes in their individual fitnesses (Fig. 6). Consider the bimodal population of PF cells with an approximately 1∶1 ratio of low and high expressors. Using live cell imaging under the microscope in a microfluidic chamber with controlled environment [67], we have tracked individual cell lineages from this population (see Fig. S12C in Text S1, and Video S1). Although cell populations were initially composed of 45% high expressors, over 30 hours the cell lineages spent 63% of the time in the high expression state. As the period of observation approaches the time of cellular memory, we expect that the cell lineages will converge to a percentage of∼97.5% in the high expression state. Thus, the population-average of gene expression measured at any time differs from the mean estimated by tracking individual cell lineages over time (Fig. 6C). The same is probably true for other statistical measures of gene expression. This is important as some research groups have been obtaining single cell-level data from large cell populations at a given time [10], [11], while other groups have been collecting gene expression data by tracking single live cells over time [74], [77]. Neither of these approaches is ideal, as both ignore information about division rates in expanding cell lineages. Our results indicate that gene expression statistics obtained from these different approaches will in general disagree, and may need to be reinterpreted in light of the fitness differences caused by gene expression.

Our work differs from earlier studies aimed to determine stochastic switching rates that optimize fitness in fluctuating environments, some of which were exclusively theoretical [37]–[39], [78], while others included experiments, but did not map the fitness landscape at increasing levels of sustained stress [23], [40], [73]. Seeking to predict optimal switching rates, these earlier studies assumed that switching rates could be altered without a fitness cost. By contrast, adjusting the memory in our system to favor of the high expression state involved an increasing fitness cost. Consequently, the switching rates that optimize fitness in our system differ from the prediction of earlier bet hedging studies, namely that maximal memory of the high expression state should optimize fitness for very long periods of drug exposure [39]. In fact, we found the exact opposite. Minimal memory of the high expression state optimized fitness in our system, as long as a minuscule high expressor subpopulation existed to salvage the population after the onset of stress. This different optimum is caused by the cost associated with increasing the memory of the high expression state. While higher memory could salvage a larger fraction of the population immediately after stress onset, it would be unfavorable on the long run due to the increased rtTA toxicity (see Section 9 and Fig. S11 in Text S1).

Is there a similar fitness cost in natural systems for increasing the memory of a stress-tolerant state? The answer appears to be “yes”. First, bistability of stress-tolerance and stress resistance states are likely wide-spread in microbes [35], due to implicit, growth rate-mediated positive feedbacks [44], [45] resulting from the expression cost or toxicity associated with various defense mechanisms. Examples include the toxin component of toxin-antitoxin systems [79], [80], *hipQ* expression for type II persisters [73], or the TetA protein from the *tetRA* tetracycline resistance operon [81]. While defense and fitness cost are caused by the same protein in these natural systems, this is not unlike our synthetic gene network, in which the sources of cost (*rtTA*) and defense (*yEGFP::zeoR*) are expressed from identical promoters, and are therefore identically regulated, - almost as if these genes were fused or were part of an operon. Increasing memory in such implicit, growth-coupled positive feedback systems implies an elevated level of toxic protein, which causes slower growth and thereby a higher fitness cost. Therefore, toxicity, toxin expression level and memory of high toxin expression are practically synonymous in these systems. It will be highly important to study at the single cell level how toxin expression coupled with toxicity controls the emergence of bistability in many microbial toxin-antitoxin systems. This was exemplified by a recent pioneering study on the *hipBA* system in *Escherichia coli* [80], where tuning the level of the HipA toxin resulted in the coexistence of normally growing and growth-arrested cells. The coexistence of such distinct phenotypes implies bistability somewhere in the network. While a growth rate-mediated positive feedback [44], [45] was not considered by [80], it would be important to investigate if its inclusion can cause bistability [82].

The potential implications of the “sweet spot” observed in our experiments are intriguing, and may relate to the extremely low fraction of persister cells observed in microbial populations [73]. If increasing the memory of the drug-tolerant state is costly, it is advantageous to dedicate as small a fraction of the population as possible to the persister state. This minute persister population will then be able to reproduce during drug treatment at a minimal fitness cost. This is true as long as persisters have extensive memory and nonzero division rates like type II persisters [73], which may decrease or vanish as the fraction of persister cells and the memory of the persister state increases. Our experimental model and computational approach can therefore be useful in studying the properties of microbial populations with small fraction of resistant cells and bet hedging strategies.

In addition, our findings have important implications for interpreting the outcome of biological measurement techniques focusing on the average gene expression in the population of cells (such as gel blot assays or microarrays). Specifically, apparent “upregulation” following stress exposure may be due to nongenetic selection of a particular pre-existing subpopulation, and may have nothing to do with actual gene regulation. For example, at the “sweet spot” Zeocin treatment caused a drastic apparent increase in average gene expression in the population that did not involve any increase in protein levels in individual cells, but was simply due to the increase of the proportion of high-expressing cells in the population. This effect was due to the selection of high expressors in the presence of Zeocin. A similar nongenetic selection mechanism may also be implicated in the emergence of microbial or cancer drug resistance, where rare pre-existing drug-tolerant cells could be selected during chemotherapy, causing additional resistance to subsequent rounds of treatment. Consistent with these studies, our results suggest that even without underlying genetic changes, gene expression variability can generate a stable, selectable subpopulation of rare survivor cells [73], [83], [84] that maintains resistance over hundreds of cell generations, and may serve as a reservoir of increasingly drug resistant mutants.

The PF circuit design can be easily extended up to mammalian systems and down to bacterial systems, due to the simplicity of the circuit design and the availability of equivalent circuit components in other organisms. Growing evidence supports the feasibility of transferring synthetic gene circuits across organisms [30], [85], which might someday enable translational applications in the clinic [70], [86]. For example, a potential application for synthetic gene circuits that consist of a self-activating regulator controlling a drug resistance gene may be engineering the microbiome [86], [87]. All parts of the human body host immense microbial communities, with cell counts that exceed by an order of magnitude the number of our own cells [88]. The microbiome can impact human physiology, and is required for vital functions of the human body. Consequently, rebalancing the microbiome may facilitate the reestablishment of homeostasis within the human body. Such rebalancing may be necessary after antibiotic treatment, which – in addition to the targeted pathogens – may drastically perturb the gut microbiome. For this reason, crucial members of the human microbiome could someday be engineered to survive or even proliferate during antibiotic treatment. Gene circuits similar to the one described above operating near the “sweet spot” may be capable of this task by ensuring near-optimal growth in both the absence and presence of antibiotic.

Another potential use of gene circuits similar to the one described above might be for *in vitro* studies of metastable states of stem cells or adult progenitor cells. It is known that these cell types can stochastically transition between various metastable states, some more prone to differentiation than others [89]–[92]. These stochastic transitions and their implications for differentiation may be difficult to study *in vivo*. On the other hand, *in vitro* conditions may drastically perturb or abolish these metastable subpopulations. Gene circuits similar to PF controlling a metastable-state specific gene (in addition to a drug resistance gene) may reestablish the desired subpopulation fractions *in vitro*. Moreover, applying selective pressure by introducing a drug into the medium could adjust the stability of these transient states independently of their subpopulation fractions. Such a system could someday provide a constant, desired yield of transient-amplifying or differentiated cells for further studies or perhaps regenerative medicine [86].

Finally, this work highlights the importance of selective environments for characterizing the behavior of synthetic gene circuits, but also has implications for studying natural gene networks. Diverse environments can broaden the functionality of regulatory modules that respond to specific external signals. Yet, understanding the environmental response of gene networks and their host cell populations is not trivial. As the size of natural and synthetic gene networks demanding quantitative description increases, a new challenge will be to describe their behavior in various well-defined environments. It will be interesting to see how quantitative modeling can tackle the combinatorial complexity associated with tuning multiple environmental factors imposed on regulatory networks.

## Materials and Methods

### Construction of plasmids

The plasmids for the construction of the PF yeast strain were created as follows. First, the *P_TETREG_* promoter consisting of two *tet*O*2* sites upstream of the minimal P_CYC1_ promoter was amplified from the pBB247 plasmid [29], [93] and inserted into the pDN-G1GZmh plasmid [42] between the *AflII* and *BamHI* sites instead of the P_GAL1-D12_ promoter resulting in the pDN-T2dGZmh plasmid. In order to facilitate the planned integration of the reporter plasmid into the *his3Δ200* locus of the YPH500 strain, a small region bearing homology to the *his3Δ200* locus was constructed by PCR and inserted in front of the *HIS3* gene between the *AhdI* and *AfeI* sites of the pDN-T2dGZmh plasmid, resulting in the pDN-T2dGZmlh plasmid. Next, a second *ADH1* terminator was removed from the pDN-T2dGZmlh plasmid, resulting in the final reporter pDN-T2dGZmxh plasmid bearing the *yEGFP::zeoR* fluorescent reporter gene under the control of the P_TETREG_ rtTA-MF inducible promoter. Next, rtTA was amplified by PCR using the pBB140 plasmid [29] as a template and inserted between the *BamHI* and *XhoI* sites of the pDN-NG1Tt plasmid [42] resulting in the pDN-NG1At plasmid. Then the P_TETREG_ promoter was cut from the pDN-T2dGZmxh plasmid and inserted into the pDN-NG1At plasmid between *AflII* and *BamHI* sites, resulting in the pDN-T2dAt plasmid. After that, the second *ADH1* terminator was removed from the pDN-T2dAt, resulting in another intermediate pDN-T2dAot plasmid. The modified version of the rtTA transactivator (rtTA-MF), consisting of the *rtetR-M2* variant amplified from pCM190-M2 [43] augmented with the short FFF activation domain [58], was created by PCR and inserted into the pDN-T2dAot between the *BamHI* and *XhoI* sites, resulting in the final pDN-T2dMFot regulatory plasmid, bearing the modified *rtTA-MF* transactivator gene under the control of the P_TETREG_ rtTA-MF inducible promoter. The *TRP1* and *HIS3* marker genes were used for all regulatory and reporter plasmids, respectively. All cloning procedures were performed in *Escherichia coli* XL-10 Gold strain (Stratagene, La Jolla, CA) using selection by ampicillin (Sigma, St. Louis, MO). All constructs were sequenced in the insert regions with double coverage. A description of the oligonucleotides used in this study can be found in Section 1 and Table S1 in Text S1.

### Strains and media

The haploid *Saccharomyces cerevisiae* strain YPH500 (*α, ura3-52, lys2-801, ade2-101, trp1Δ63, his3Δ200, leu2Δ1*) (Stratagene, La Jolla, CA) was used as a model organism throughout this study. A modified lithium acetate procedure was used for transformation [94]. Only the transformed strain with a single copy of the PF gene construct was used, as confirmed by PCR. Cultures were grown in synthetic drop-out medium with the appropriate supplements to maintain selection (all reagents from Sigma, St. Louis, MO) and supplemented with sugars.

### Cell cultures and flow cytometry

Yeast strain colonies were picked from plates and incubated overnight in synthetic drop-out medium supplemented with 2% glucose at 30°C. On the next day, cell suspensions were washed to remove glucose and diluted at the concentration 4×10^6^ cells/ml in the fresh medium to produce starter cultures. Then, 100 µl was used to inoculate synthetic drop-out medium supplemented with 2% galactose and increasing concentrations of ATc (ACROS Organics, Geel, Belgium). Cultures were then allowed to grow at 30°C for at least 48 hours, to stabilize the expression in the population and then read on the FACScan flow cytometer (Becton Dickinson, Franklin Lakes, NJ). In the experiments where cultures were maintained over several days, cells were resuspended every 12 hours to keep them in the log growth phase.

### Flow cytometry data processing and analysis

We applied a small gate to minimize the contribution of extrinsic noise due to cell cycle phase, cell size and age on our analysis. A two-dimensional Gaussian fit was performed on the log-values of side scatter and forward scatter data for all stabilized dose response experiments. An elliptical gate corresponding to 90% of the maximal probability of the fit two-dimensional Gaussian distribution was applied to all data sets.

### Custom bimodality test applied to gene expression distributions

The number of events for an individual bin in the flow cytometry histograms is nearly Poissonian [95], [96]. Data was smoothed with a 32-point moving average to estimate the expected number of events for each fluorescence bin. This smoothed data was assumed to have a normal distribution with variance equal to the expectation divided by 32 (corresponding to 32 moving average). Bimodality was assessed by determining if minima located between two maxima were more than 4 standard deviations below both maxima, using a standard z test such that 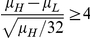, where *μ_H_* is the maximum height of the smoothed events and *μ_L_* is the minimal height of smoothed events to be tested. A more detailed description can be found in Section 2 in Text S1.

### Classification of cell populations into low and high expressors

Sub-populations in dose-response experiments were identified by using the custom bimodality test. Multiple minimal thresholds were filtered by choosing the threshold that maximized the product of the two z-scores. The threshold was found to be ∼50 fluorescent a.u. for PF cells at 10 ng/ml ATc. Subsequently, experimental data obtained from the fluorescence-activated cell sorting experiments were classified into sub-populations using a constant threshold of 50 fluorescent a.u. for PF cells. A more detailed description can be found in Section 2 in Text S1.

### Measures of gene expression variability

We calculated the mean *μ_p_* and the coefficient of variation *CV_p_* for individual cell populations, *p* based on gated FL1 (fluorescence intensity) values of cells from the corresponding culture, according to the formulae
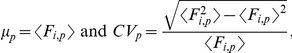
where *F_i_*_,*p*_ represents the fluorescence intensity of cell *i* from population *p*, while the angled brackets denote averaging over individual cells, *i* from population *p*.

### Purification of high expressor cells by serial dilution

We preinduced and repeatedly resuspended a PF cell population for 4 days in 10 ng/ml ATc to obtain a stable bimodal expression pattern. Next, we estimated the cell concentration using a NexCelom Cellometer T4 and prepared a set of 80 separate 1 ml cultures using multiple serial dilutions, aiming to obtain approximately 1 cell per 10 tubes. After repeatedly resuspending these diluted cultures into the same 10 ng/nl concentration of ATc inducer, we measured the resulting gene expression distributions on day 4.

### Fluorescence microscopy

We preinduced and repeatedly resuspended a PF cell population for 4 days at an appropriate inducer concentration to obtain a stable bimodal gene expression pattern. Phase-contrast and fluorescence images were then recorded every 30 minutes over 3 days in a microfluidic chamber with continuous supply of medium containing the same inducer concentration.

### Fitting the population dynamics model to the experimental data

The set of differential equations for the two state population dynamic model ([6]) has the analytical ratio of low to high expressor cells given by
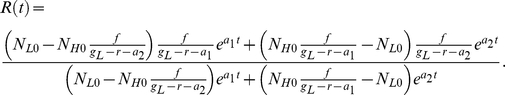
with the corresponding eigenvalues
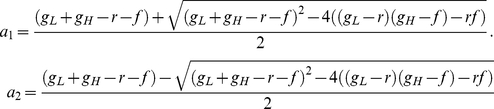
The resulting log transformed ratio ln[*R*(*t*)] = ln[*N_L_*(*t*)/*N_H_*(*t*)] was fit to the measured log-ratios of low to high expressor subpopulations using Matlab's fminsearch function based on the Nelder-Mead fitting algorithm (Fig. 4E). The data was normalized by dividing the sorted log-ratio at each time point by the corresponding log-ratio of the unsorted population, and then multiplying by the mean of all unsorted log-ratios over all time points (see Section 8 in Text S1 for details). The overall cell division rate after long periods of time (asymptotic cell division rate) is *g_T_* = *a_1_*.

### Fitting the overall population fitness function to experimental data

Instantaneous fitness in Zeocin depended on the balance of cellular Zeocin resistance (approximated by the fluorescence level) and extracellular Zeocin. Zeocin toxicity was modelled by focusing on the DNA which transitioned to a damaged state when bound by Zeocin, and was repaired at a constant rate. The Zeocin fitness function was defined as
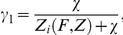
where *Z_i_* is Zeocin concentration within the cell and *χ* is a constant related to intracellular Zeocin toxicity. Zeocin was as assumed to diffuse into and out of the cell freely, and to bind irreversibly to yEGFP::ZeoR. Intracellular Zeocin concentrations were modeled by mass action kinetic reactions at steady state:
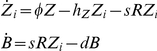
where *h_Z_* = 0.5, *d* = 0.25, while *Z*, *B*, and *R* are external Zeocin, and bound and unbound yEGFP::ZeoR concentrations. The total fluorescence constrains yEGFP::ZeoR by the equation *F* = *R*+*B*. The rate constants are *h_Z_* (Zeocin diffusion out of the cell membrane), *s* (yEGFP::ZeoR binding affinity for Zeocin), and *d* (yEGFP::ZeoR degradation/dilution rate, assumed to be constant for simplicity). A second function describing the instantaneous reduction in PF fitness as a function of fluorescence and ATc was defined as
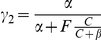
where *F* is the fluorescence value of the cells, and *C* is the ATc concentration cells are grown in. The parameter *α* describes the toxicity of activated rtTA, and *β* describes the binding efficiency of rtTA to ATc.

The parameters we obtained after fitting were: *φ* = 1.182, *χ* = 0.5028×10^−7^, *s* = 1.2732×10^6^, *α* = 936, *β* = 5.8.

### Estimating cellular currents with fitness corrections

Cellular currents were defined by the functions
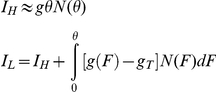
where *g*(*F*) is the instantaneous fitness at fluorescence *F*, and *g_T_* is the overall fitness of the cell population. Integrals were numerically calculated based on the fitness function and flow cytometry distributions using Matlab's *trapz* function.

## Supporting Information

Text S1 **Details of experimental, theoretical and computational methods used in this study.** Text S1 contains: the list of primers used in this study for constructing the PF gene circuit; the description of the custom bimodality detection algorithm; various mathematical modeling approaches for determining the dynamic behavior and cellular memory of the PF synthetic gene circuit; the derivation of the formulas for instantaneous cellular fitness in various conditions; the derivation of the cellular current and its relationship to cellular memory; the details of experimental cellular memory measurements; the description of why the Kussell-Leibler optimality predictions are invalid for our experimental system; stochastic simulations accounting for differential cell division; and the results of tracking individual cells in Video S1.
(PDF)

Video S1 **Gene expression dynamics of growing and dividing PF cells.** Genetically identical *S. cerevisiae* cells carrying the chromosomally integrated PF gene circuit imaged every 30 minutes over 2 days while maintained at constant temperature (30 C) and constant, intermediate inducer concentration. The movies consist of overlays of individual phase-contrast and fluorescence (EPI) images recorded in the FITC channel on a Nikon TiE microscope using NIS Elements Advanced Research.
(AVI)
