# Supplementary material for: Mapping the Environmental Fitness Landscape of a Synthetic Gene Circuit
Source: PLoS Comput Biol. 2012 Apr 12;8(4):e1002480. doi: 10.1371/journal.pcbi.1002480 (PMC3325171; doi:10.1371/journal.pcbi.1002480)
Supplement: Text S1 — Details of experimental, theoretical and computational methods used in this study. Text S1 contains: the list of primers used in this study for constructing the PF gene circuit; the description of the custom bimodality detection algorithm; various mathematical modeling approaches for determining the dynamic behavior and cellular memory of the PF synthetic gene circuit; the derivation of the formulas for instantaneous cellular fitness in various conditions; the derivation of the cellular current and its relationship to cellular memory; the details of experimental cellular memory measurements; the description of why the Kussell-Leibler optimality predictions are invalid for our experimental system; stochastic simulations accounting for differential cell division; and the results of tracking individual cells in Video S1. (PDF) [file pcbi.1002480.s001.pdf]

# Text S1

# Mapping the Environmental Fitness Landscape of a Synthetic Gene Circuit

Dmitry Nevozhay, Rhys M. Adams, Elizabeth Van Itallie, Matthew R. Bennett, and Gábor Balázsi

**1. Primers used in this study ... 2**
**2. Collection and processing of flow cytometry data ... 2**
*2.1. Preprocessing of flow cytometry data ... 2*
*2.2. Detecting gene expression bimodality and classifying cells as high or low expressors ... 3*
*2.3. Elimination of outliers ... 4*
**3. Deterministic mathematical model of cells carrying the PF gene construct ... 4**
**4. Using escape rate theory to estimate cellular memory ... 5**
*4.1. Calculating the Mean First Passage Time (MFPT) ... 5*
**5. Overall cell population fitness and instantaneous cellular fitness ... 7**
*5.1. Instantaneous cellular fitness reduction due to Zeocin toxicity ... 8*
*5.2. Instantaneous cellular fitness reduction due to rtTA-associated toxicity ... 8*
*5.3. The combined instantaneous cellular fitness ... 10*
**6. Using the cellular current for estimating cellular memory with differential fitness ... 11**
*6.1. Calculating the downward cellular current for the PF gene circuit ... 13*
*6.2. Calculating the upward cellular current for the PF gene circuit ... 14*
*6.3. Estimating the cellular memory based on the cellular current ... 14*
*6.4. Estimating the error from cellular current ... 15*
*6.5. Cellular memory estimations for different gene circuits and inducer concentrations ... 15*
**7. Fitness measurements ... 15**
*7.1. Measuring the resuspension period for keeping PF cells in the exponential growth ... 15*
*7.2. Fitness measurements of PF cells maintained in exponential growth ... 16*
**8. Switching rate measurements based on the two state population-dynamics model ... 16**
*8.1. Estimating the non-genetic memory of high and low expressing cells by sorting bimodal cell populations ... 16*
*8.2. Using purified subpopulations of high-expressing PF cells to estimate cellular memory ... 18*
**9. Ideal phenotypic switching rates ... 18**
*9.1. Mutual dependence of switching rates and fitness affects optimal survival strategies ... 18*
*9.2. The "sweet spot" occurs when switching rates are less than growth rates ... 19*
**10. Gillespie simulations of cell populations maintained in exponential growth ... 19**
**11. Tracking cells from micoscopic images obtained in a microfluidic device ... 20**

## 1. Primers used in this study

The primers Tetreg-AflII-f, CYC1-BamHI-r, T2-Down-f and T2-Up-r were used to repair the second *tetO2* site and amplify the $P_{TETREG}$ promoter from the pBB247 plasmid [1,2]. Five primers (Amp-AhdI-f, HIS3-Add1-r, HIS3-Add2-r, HIS3-Add3-r and HIS3-AfeI-r) were used to construct a small region homologous to the *his3Δ200* locus in the pDN-T2dGZmh plasmid. The Origin-PstI-f and Origin-SacI-r primers were used to amplify a region without the *ADH1* terminator and insert it into the pDN-T2dGZmxh plasmid. Primers Tetreg-AflII-f, Second-tetO-f, CYC1-r, HISSeq-r, Origin-Pst-f, HISEnd-f, TADH-r, Origin-middle-f and GalSeqE-r were used for sequencing.

Two primers, rtTABamHI2-f and VP16XhoI-r were used to amplify rtTA from the pBB140 plasmid [1]. The shorter fragment without the *ADH1* terminator sequence was amplified using the TRP-f and Origin-AflII-r primers. The primer rtTA-BamHI2-f was used with two other primers (FFF-Add-r and FFF-XhoI-r) in sequential PCR reactions to add a short FFF activation domain [3] to the rtTA-M2 variant [4]. The plasmids TRP-f, TRP-r, Origin-middle-f, CYC1-BamHI-r, Tetreg-AflII-f, rtTA-BamHI2-f, FFF-XhoI-r, Backbone-r and Origin-AflII-r were used to sequence the intermediate pDN-T2dAot and the final pDN-T2dMFot regulatory plasmids.

## 2. Collection and processing of flow cytometry data

### *2.1. Preprocessing of flow cytometry data*

Population-level statistics of fluorescence intensities in single yeast cells were obtained using flow cytometry. During each run, ~50,000 cells were measured to obtain their forward scatter (FSC), side-scatter (SSC), and fluorescence intensity (FL1) values, which were classified into logarithmic bins of three histograms. Considering that the FSC value correlates with cell size while the SSC value correlates with cell granularity, we filtered the data considering only cells within a narrow gate in the [ln(FSC), ln(SSC)] space, to minimize the effects of "extrinsic" noise originating from variations in cell size or age.

Ideally, the gate used to reduce extrinsic noise should have minimal area while maximizing the number of measured events for each sample. This requires for the gate to be centered at the region of highest cell density within the [ln(FSC), ln(SSC)] space. In addition, we aimed to collect fluorescence measurements of similar-sized cells from various samples. To achieve this, we established and maintained a fixed elliptic gate for all fluorescence measurements based on the observation that the FSC-SSC scatter plots were consistently similar throughout all inducer concentrations for both strains. Therefore, we pooled [ln(FSC), ln(SSC)] values from cells grown at various ATc concentrations, and then we fit a two-dimensional normal distribution to the pooled ln(FSC)-ln(SSC) histogram using the "*gmdistribution.fit*" function in Matlab. The resulting two-dimensional normal distribution had [ln(FSC), ln(SSC)] means of $[\mu_1 \quad \mu_2] = [4.8 \quad 4.1]$ and a covariance matrix of $\begin{bmatrix} \sigma_{11} & \sigma_{12} \\ \sigma_{21} & \sigma_{22} \end{bmatrix} = \begin{bmatrix} 0.21 & 0.22 \\ 0.22 & 0.34 \end{bmatrix}$, where the subscript "1" corresponds to ln(FSC), while the subscript "2" corresponds to ln(SSC). Finally, we defined the gate by the boundary where this normal distribution reached 90% of its maximum value. On average 5168±1681 (but always more than 1500) cells were collected within the fixed elliptic gate during each measurement.

### *2.2. Detecting gene expression bimodality and classifying cells as high or low expressors*

A crucial component of our data analysis was detecting bimodality of fluorescence distributions measured by flow cytometry. Because some of our fluorescence data exhibited skewed, non-normal modes, detecting bimodality using Gaussian mixture models was problematic [5]. Defining bimodality based on a local minimum in fluorescence histograms between two maxima was difficult as well, since the number of cells in each fluorescence bin was subject to Poissonian noise [6].

We classified a cell population as having bimodal expression if the fluorescence histogram had a local minimum between two distribution maxima, or "peaks". However, variations in the numbers of cells classified within a bin during flow cytometry measurements could result in false positives since noisy cell counts could also result in local minima. Thus, we used statistical tests to rigorously distinguish significantly bimodal distributions from distributions whose apparent bimodality was due to random fluctuations from flow cytometric binning of cells.

Gated fluorescence intensity histograms were first smoothed with a 32-point moving average to estimate the expected number of events for each fluorescence bin. Since the number of events for a bin is approximately Poissonian [6,7], we expect the variance for the number of events within each bin to be equal to the average number of events. Furthermore, since the *average* number of events is obtained by smoothing, the Central Limit Theorem states that this smoothed data should tend towards a normal distribution whose standard deviation is equal to the number of cells detected, divided by the square root of the number of points used for smoothing (i.e. $\sqrt{32}$ ).

Given two local maxima of heights $h_L$ and $h_R$, located to the (*L*)eft and (*R*)ight of a local (*M*)inimum of height $h_M$, we rejected the null hypothesis that the distribution was unimodal if $h_M$ was at least 4 standard deviations (estimated at each local maximum) lower than both $h_L$ and $h_R$,

$$\begin{aligned} &|h_M - h_L| > 4\sigma_L = 4\sqrt{h_L/32} \Leftrightarrow z_L > 4 \\ &AND \qquad . \\ &|h_M - h_R| > 4\sigma_R = 4\sqrt{h_R/32} \Leftrightarrow z_R > 4 \end{aligned} \qquad [1]$$

In the dose-response measurements, the cutoff used to separate high- and low expressor subpopulations for any individual histogram was defined as the location of the local minimum (see for example Figure S1, where the bimodality detection algorithm was applied to yeast cells carrying a different gene circuit, with less distinct fluorescence peaks). If multiple significant minima were observed, then we selected the minimum with the highest product of z-scores,

$$\max(z_L \times z_R), \qquad [2]$$

as the adaptive cutoff for separating low and high expressor subpopulations.

For experimental cellular memory measurements based on relaxation towards a stationary distribution, following fluorescence-activated cell sorting (FACS) of cells into high and low expressors, we used constant cutoffs to classify cells into low and high expressor subpopulations. The cutoffs were chosen as 50 arbitrary fluorescence units (a.u.) for PF cell populations. The

relaxation of the subpopulation ratio $R(t)=N_L/N_H$ to its asymptotic value was followed in time to estimate stochastic switching rates between high and low yEGFP expression.

### 2.3. Elimination of outliers

Flow cytometry measurements may involve a small number of contaminating "events" due to mutated cells that have lost the integrated PF construct (due to homologous recombination), or due to rare leftover cells from previous samples which may not have been completely eliminated from the flow cytometer. A small population of such contaminants could strongly affect standard deviation-based measures, such as the coefficient of variation (CV). Thus, we eliminated distant outliers in all flow cytometry measurements by two different methods depending on whether the gene expression was bimodal or not. For unimodal populations, we eliminated cells more than 3 standard deviations above and below the mean of the log-transformed data. For *bimodal* populations we eliminated cells more than 3 standard deviations below the mean of the low peak in the log-transformed FL1 data, as well as cells more than 3 standard deviations above the mean of the high peak in the log-transformed FL1 data.

## 3. Deterministic mathematical model of cells carrying the PF gene construct

The PF gene circuit was modeled using a system of ordinary differential equations (ODEs) [8,9]

$$\begin{aligned}
\dot{w} &= aF(x) + l - bwy - (\delta + g)w \\
\dot{x} &= bwy - (\delta + g)x \\
\dot{y} &= C - bwy - hy \\
\dot{z} &= aF(x) - gz + l
\end{aligned} \qquad [3]$$

where $F(x) = x^n/(\varphi^n + x^n)$, and the variables $w$, $x$, $y$ and $z$ correspond to inactive and active intracellular TetR, inducer and reporter concentrations, respectively, while $C$ is a control parameter proportional to the extracellular inducer concentration. Other parameters are $a$ (protein synthesis rate), $b$ (inducer-repressor association rate), $\delta$ (degradation rate), $g$ (rate of dilution due to cell growth), and $h$ (combined rate of inducer dilution, outflux and degradation). Whenever possible, parameters in these equations were obtained from the literature, while the remaining unknown parameters were estimated by fitting the ODEs to the experimentally observed sub-population means.

Using this set of parameters, we studied the dynamic behavior of the system described by the system of ODEs [3]. Considering that $z$ does not affect any of the other variables, it is sufficient to analyze the first three ODEs. Using the notation $v=x+w$, after adding the first two ODEs, and considering that at most ATc concentrations overall protein synthesis and degradation are rate-limiting processes, the second and third equations equilibrate at relatively fast time scales to give:

$$\begin{aligned}
\dot{v} &= aF(x) + l - (\delta + g)v \\
0 &= C\frac{b(v-x)}{b(v-x)+h} - (\delta + g)x
\end{aligned} \qquad [4]$$

from where, considering the only physically meaningful solution of a quadratic equation $x$ can be expressed as a function of $v$ as

$$x(v)=\frac{1}{2}\left(\frac{C}{\delta+g}+\frac{h}{b}+v\right)-\frac{1}{2}\sqrt{\left[\frac{C}{\delta+g}+\frac{h}{b}+v\right]^2-4\frac{Cv}{\delta+g}}\ . \qquad [5]$$

Expressing the differential equation in terms of synthesis and degradation terms,

$$\dot{v}=k_+(v)+k_-(v)=aF[x(v)]+l-(\delta+g)v \qquad [6]$$

The intersection points of the curves $k_+(v)$ and $k_-(v)$ correspond to the steady states of the system. We can now apply rate balance analysis [10] to determine the stability of these steady states. Varying the parameter $C$, we observe that the system undergoes a saddle-node bifurcation where a single stable steady state splits into three steady states, two of which are stable, while the third is unstable (see Figure S2).

## 4. Using escape rate theory to estimate cellular memory

Intuitively, we expected the PF construct to have a strong non-genetic memory of the high expression state due to bistability, whereby the incorporation of a positive feedback loop coupled with a nonlinear promoter response exaggerates the stability of cells staying in either low expression states or high expression states. Consequently, low expressors do not produce transcriptional activator, while high expressors produce transcriptional activators at an elevated rate, in agreement with the understanding that positive feedback favors bimodal gene expression [1,11].

### *4.1. Calculating the Mean First Passage Time (MFPT)*

The first method we applied to estimate the memory of the low and high *yEGFP::zeoR* expression states was based on escape rate theory. We calculated the mean first passage time (MFPT) across a preset threshold [12,13] of regulator expression, after converting the set of ODEs [7] into Fokker-Planck equations (see below). The equations used to calculate the MFPT based on steady-state solutions (distributions) of the Fokker-Planck equations for the PF strain are given below, while the corresponding parameters are listed in Table S2.

We defined the mean first passage time (MFPT) as

$$MFPT=\frac{\int_0^{v_b} p_s(v)\tau(v,v_b)dv}{\int_0^{v_b} p_s(v)dv} \qquad [8]$$

where $\tau(v, v_b)$ is the first passage time from the starting molecular concentration $v$ to the boundary concentration $v_b=\theta$ and $p_s(v)$ is the stationary probability of a cell having an initial rtTA concentration of $v$. The parameter $v$ denotes total rtTA concentration ($x+w=v$) for PF cells.

The stationary probability distribution $p_s(v)$ (Figure S3A) was obtained using the Fokker-Planck approximation of the Chemical Master Equation [14], defined as

$$0 = -\frac{\partial}{\partial v}\left[\mu(v)p_s(v)\right] + \frac{1}{2}\frac{\partial^2}{\partial v^2}\left[\sigma(v)p_s(v)\right], \qquad [9]$$

where $\mu$ is the deterministic "mean" drift coefficient and $\sigma$ is the stochastic diffusion coefficient as a function of the molecular species concentration $v$. Knowing $\mu$ and $\sigma$, the Fokker-Planck equation [9] can be formally solved and the corresponding solution can be numerically estimated based on the equation

$$p_s(v) = \frac{Ae^{-U}}{\sigma(v)}, \qquad [10]$$

where

$$U = -2\int\frac{\mu(v)}{\sigma(v)} = -2\int\frac{k_+(v) - k_-(v)}{k_+(v) + k_-(v)} \qquad [11]$$

fulfills the role of a type of chemical potential.

The Dynkin equation [12],

$$-1 = \mu(v)\frac{\partial}{\partial v}\left[\tau(v,\theta)\right] + \frac{1}{2}\sigma(v_0)\frac{\partial^2}{\partial v^2}\left[\tau(v,\theta)\right], \qquad [12]$$

was then used to obtain the first passage time (Figure S3B) for cells with a starting concentration $v_0$ to arrive at the boundary concentration $\theta$, denoted $\tau(v,\theta)$.

Using quasi-steady state approximations of [3], we approximate the drift $\mu_{PF}$ and diffusion $\sigma_{PF}$ functions for total rtTA concentrations ($v = x + w$) as

$$\begin{aligned} \mu_{PF} &= k_+ - k_- = aF(x) - (\delta + g)v \\ \sigma_{PF} &= k_+ + k_- = \left(aF(x) + (\delta + g)v\right)/\Omega \end{aligned}, \qquad [13]$$

where $\Omega$ denotes the cell volume. Active rtTA levels ($x$) are given as a function total rtTA ($v$) by equation [5].

Having defined the MFPT in terms of the Dynkin equation, we asked if we could apply a yet simpler analytical approach to calculate cellular memory.

Kramer's escape rate theory treats stable states as potential wells that can be approximated by a quadratic function at the extrema of the chemical potential [15-17]. Approximating the potential around extrema as a second order Taylor expansion results in:

$$\begin{aligned} V(F) &\approx V(v_{min}) + \frac{1}{2}V''(v_{min})(v - v_{min})^2 \\ V(F) &\approx V(v_{boundary}) - \frac{1}{2}\left|V''(v_{boundary})\right|(v - v_{boundary})^2 \end{aligned} \qquad [14]$$

where

$$V(v) = -\int_0^v \mu_{PF}(v)dv\,, \qquad [15]$$

and $v_{min}$ is a stable steady state of the system, and $v_{boundary}$ is an unstable steady state defining the boundary condition (similar to $\varphi$).

When the diffusion term ($\sigma$) is practically constant, $U(v)$ is approximately

$$U(v) \approx \frac{V(v)}{\sigma}. \qquad [16]$$

The MFPT [8] can then be approximated as

$$\tau = \ln(2)\frac{2\pi}{\sqrt{|V''(v_{min})||V''(v_{boundary})|}}\exp\left[\frac{V(v_{boundary}) - V(v_{min})}{\sigma(v_{min})}\right]. \qquad [17]$$

Applying this formula to the PF equations (Table S2, eqn [13]) gave the MFPT predictions in figure S3C.

**5. Overall cell population fitness and instantaneous cellular fitness**

Fitness is an important phenotype that describes the rate of increase in the relative number of individuals carrying a certain genotype. The source of increase in cell population size is the division of individual cells. Therefore, fitness measured at the cell population level (by competition experiments or population growth assays) must be dependent on the time necessary for individual cell division events. There is a tacit assumption that population-level measurements represent the typical rate of cell division, which is uniform across the population. However, this assumption is not always true. Just like the average of gene expression does not always represent any typical cell, the overall growth rate of a cell population may not always represent the division time of any individual cell. This is particularly true for PF cells that differentiate into states characterized by very different gene expression levels and cell division times. To properly establish the connection between individual cell division times and cell population growth, we define two types of fitness: *instantaneous cellular fitness* and *overall cell population fitness*. The first (instantaneous cellular fitness) is defined as the inverse of the time of a full cell cycle for cells with gene expression level $v$.

$$g(v) = T^{-1}_{cell\ cycle}(v)$$

The second type of fitness (*overall cell population fitness*) is the rate of increase of the relative population size, or the change in the total number of cells over time ($\dot{N}_T$) divided by the total number of cells ($N_T$),

$$g_T = \frac{\dot{N}_T}{N_T}.$$

The connection between the instantaneous cellular fitness and overall cell population fitness can be expressed as:

$$g_T = \int_0^\infty g(v)p(v)dv, \quad [18]$$

where $p(x)$ is the probability density function of cells at the state $x$. That is, $g_T$ is the arithmetic average of the instantaneous fitness weighted by the probability distribution of cellular states.

### *5.1. Instantaneous cellular fitness reduction due to Zeocin toxicity*

We modelled Zeocin toxicity as DNA transitioning to a damaged state due to Zeocin binding and inducing DNA strand breaks. We also assumed that DNA damage repair occurred at a constant rate faster rate than cell division time, so that the instantaneous fitness reduction was proportional to the fraction of time that DNA remained undamaged. The quasi-stationary solution for the instantaneous fitness reduction due to DNA damage was defined as

$$\gamma_1 = \frac{\chi}{Z_i(F,Z) + \chi}, \quad [19]$$

where $Z_i$ is the intracellular Zeocin concentration and $\chi$ is the rate of DNA repair divided by the rate of Zeocin-induced damage accumulation.

To determine $Z_i$, Zeocin was assumed to diffuse into and out of the cell, and bind irreversibly to yEGFP::ZeoR. Intracellular Zeocin concentration was modeled by mass action kinetics:

$$\begin{aligned} \dot{Z}_i &= Z - h_Z Z_i - sRZ_i \\ \dot{B} &= sRZ_i - dB \end{aligned} \quad [20]$$

where $Z$, $B$, and $R$ are external Zeocin, Zeocin-bound and free yEGFP::ZeoR concentrations, respectively. The total fluorescence $F$ imposes the constraint $F{=}R{+}B$. The rate constants are $h_Z$ (Zeocin diffusion out of the cell membrane), $s$ (yEGFP::ZeoR binding affinity for Zeocin), and $d$ (yEGFP::ZeoR degradation/dilution rate, assumed to be constant for simplicity). Assuming that free Zeocin equilibrates quickly, we obtained the intracellular Zeocin concentration from [20] as

$$Z_i = \frac{ksZ - dh_Z - dsF + \sqrt{(ksZ - dh_Z - dsF)^2 + 4h_Z sdkZ}}{2h_Z s}. \quad [21]$$

### *5.2. Instantaneous cellular fitness reduction due to rtTA-associated toxicity*

There are several sources of toxicity that cells may experience when their rtTA and yEGFP::zeoR expression levels are high. Toxicity may come from the rtTA protein itself due to the so-called "squelching" effect, due to rtTA's VP16 activation domain, which can sequester general factors involved in eukaryotic gene expression, such as ADA2, TFIIB, TFIID, etc – see [3] and references therein. In addition to these earlier findings, we propose a specific fitness decrease due to the ATc-bound form of rtTA. We have three lines of evidence that support this proposal.

First, we compared uninduced PF cells (no rtTA) to cells with cells constitutively expressing rtTA (PR cells, Figure S5A) in the absence of ATc. As expected, we noted a drop in fitness of PR cells due to constitutive rtTA expression (see Figure S5C). Subsequently, we compared the fitness of PF and PR cells in the absence and presence of saturating ATc concentrations (200 ng/ml) to determine the effects of free and ATc-bound rtTA proteins. We noted an additional decrease in fitness for PR cells grown in 200 ng/ml ATc (compared to PR cells grown in 0 ng/ml ATc). Since rtTA expression in this strain is constitutive, total rtTA expression is constant and independent of ATc. Taken together with the fitness decrease at 200 ng/ml ATc, this suggests that the ATc-bound form of rtTA had stronger toxicity than rtTA alone (see Figure S5C).

Second, we created two alternative models of rtTA toxicity and used them to predict the overall cell population fitness in 2 mg/ml Zeocin. The first alternate model assumed that fitness was best described as a decreasing function of <u>total</u> *rtTA* expression, given as:

$$\gamma_2(F,C) = \frac{\alpha}{\alpha + F}. \qquad [22]$$

The second alternative model separately attributed toxicity to both forms of rtTA (active and inactive). These rtTA forms had different toxicities, as modeled by the equation:

$$\gamma_2(F,C) = \frac{1}{1 + \alpha^{-1} \underbrace{F \frac{C}{C+\beta}}_{\text{Active rtTA}} + \kappa^{-1} \underbrace{F \frac{\beta}{C+\beta}}_{\text{Inactive rtTA}}}. \qquad [23]$$

Both models were able to account for the general trends of overall cell population fitness (i.e., presence of "sweet spot") in the presence of Zeocin when memory was taken into account (see Figure S5D and E). However, consistent with observations of PF fitness, we found that active ATc-bound rtTA was the variable most predictive of overall cell population fitness in new environments.

The third and final evidence comes from the literature. ATc binding to rtTA should affect the DNA-binding ability of this activator, suggesting that rtTA toxicity increases upon its binding to DNA. For the VP16 activator domain in another chimaeric construct it was shown that mutating the DNA-binding domain of the protein fusion abrogated activator toxicity [18].

In addition to the toxicity directly attributable to the rtTA protein itself, cells in the high expressing state may experience additional, indirect toxicity due to the costs of rtTA and yEGFP::zeoR transcription and translation [19,20]. To test if this was the case we performed an additional experiment using a strain called PFX (Figure S5B), which was identical with PF, except that it lacked the *yEGFP::zeoR* coding region (Figure 2A). We found that the fitness

reduction in PFX cells at large concentrations of ATc was smaller than that for PF cells, suggesting that the transcription and/or translation in the reporter gene are metabolically costly and further augment rtTA toxicity in high expressor cells (Figure S5F).

Without knowing the exact contributions of intracellular rtTA toxicity (squelching, transcriptional and translational burdens), we may nonetheless define the instantaneous cellular fitness reduction, $\gamma_2(x,C)=g(x,C)/g_0$ phenomenologically through the Hill function [11]

$$\gamma_2 = \frac{\alpha}{\alpha + x}, \qquad [24]$$

where $\alpha$ is a constant that relates active rtTA ($x$) concentration to activator toxicity. Because active rtTA ($x$) concentration cannot be measured directly, we estimated it from the fluorescence concentration ($F$) and ATc ($C$), using the approximation $b(v\text{-}x)C=dx$ as

$$x \propto F\frac{C}{C+\beta}, \qquad [25]$$

where fluorescence ($F$) is a proxy for total rtTA, and the fit parameter $\beta$ represents the efficiency of ATc-rtTA binding. Assuming that the main source of toxicity is ATc-bound rtTA, the instantaneous fitness reduction can be defined from [24] and [25] as

$$\gamma_2(F,C) = \frac{\alpha}{\alpha + F\frac{C}{C+\beta}}. \qquad [26]$$

### *5.3. The combined instantaneous cellular fitness*

Considering that different mechanisms underlie the toxicities of rtTA and Zeocin toxicity (sequestration of general transcription factors and DNA damage, respectively), we assumed Bliss independence [21] and calculated the combined instantaneous fitness reduction due to rtTA and Zeocin toxicity as the product of their individual effects:

$$g(F,C,Z) = g_0 \times \frac{\chi}{Z_i(F,Z)+\chi} \times \frac{\alpha}{\alpha + F\frac{C}{C+\beta}}. \qquad [27]$$

The Loewe model of drug additivity can be used alternatively to the Bliss independence model. In contrast to the Bliss independence assumptions of different mechanisms of toxicity, Loewe additivity assumes that toxic molecules act on the same biological site, but differ in their toxicity[22]. Loewe additivity takes the form:

$$g(F,C,Z) = g_0 \times \frac{\alpha\chi}{\alpha\chi + \alpha Z_i(F,Z) + \chi x(F,C) + \iota Z_i(F,Z)x(F,C)}. \qquad [28]$$

where x(F,C) was previously defined ([25]) and $\iota$ is the interactivity index. For negative $\iota$ the toxic molecules are antagonistic, for positive $\iota$ the toxic molecules are synergistic. We created an alternative set of fitness landscape predictions for antagonistic interactions ($\iota$=-100), neutral interactions ($\iota$=0), and synergistic interactions ($\iota$=100 to 500) as shown in Figure S6G and H [23]. There were almost no qualitative changes between the different models incorporating memory—as synergism increased the fitness decreased, but the curve consistently peaked at 1 ng/ml ATc at 2 mg/ml Zeocin, and slowly declined as ATc was increased.

**6. Using the cellular current for estimating cellular memory with differential fitness**

Stationary fluorescence distributions of growing cell populations do not imply the lack of fluorescence changes in individual cells. On the contrary, random and regulated gene expression changes in single cells induce their constant movement within such distributions. Specifically, upward cellular movement within the distribution corresponds to increasing intracellular fluorescence, while downward movement corresponds to decreasing intracellular fluorescence. To characterize cellular movement and division within stationary distributions, we introduce the concept of *cellular current*, which is somewhat analogous to the electric current through a preset cross-section within a cable.

We define *directional cellular currents* as the number of cells ($N_{H\to L}$, and $N_{L\to H}$) that move in a given direction (leftward and rightward within the fluorescence distribution, respectively) through a preset fluorescence concentration threshold ($F$) per unit time:

$$I_{H\to L}(F)=\frac{N_{H\to L}(F,\Delta t)}{\Delta t} \text{ and } I_{L\to H}(F)=\frac{N_{L\to H}(F,\Delta t)}{\Delta t}, \quad [29]$$

where the "fluorescence concentration" is defined by the relationship

$$F=\frac{P}{\Omega_F}, \quad [30]$$

where $\Omega_F$ is a parameter relating intracellular yEGFP::ZeoR protein copy number ($P$) to measured fluorescence "concentrations".

The *net cellular current* is the difference of directional cellular currents:

$$I(F)=I_+(F)-I_-(F). \quad [31]$$

Consider a stationary distribution with uniform instantaneous growth rates $g(F)$ that are equal to the overall growth rate $g_T$ (flat nongenetic fitness landscape). Every region enclosed by fluorescence values ($F_1$,$F_2$) in such a distribution will increase at rate $g_T N(F_1,F_2,t)$, where $N(F_1,F_2,t)$ is calculated via the integral:

$$N(F_1,F_2,t)=\int_{F_1}^{F_2}\frac{\partial N(F,t)}{\partial F}dF=N_T\int_{F_1}^{F_2}p(F,t)dF, \quad [32]$$

while $\partial N(F,t)/\partial F$ is the local cell density estimated at time $t$, as the overall cell number $N_T$ multiplied by the probability density function of fluorescence $p(F,t)$.

While random movement in- and out of such regions is possible, the cellular current *exiting* the region enclosed by fluorescence values $F_1<F_2$, must equal 0 (or else the distribution would be reshaped). This is expressed by the *continuity equation*:

$$\Delta I(F_1, F_2) = I(F_2) - I(F_1) = 0\,, \qquad [33]$$

which implies that the net currents $I(F_1)$ and $I(F_2)$ are equal:

$$I(F_2) = I(F_1)\,. \qquad [34]$$

If the two fluorescence values $F_1$ and $F_2$ approach each other, the above equation implies that the *gradient of the net cellular current* must be equal to 0:

$$\Delta I(F, F+\Delta F) = I(F+\Delta F) - I(F) \approx \frac{\partial I}{\partial F}\Delta F = 0 \Rightarrow \frac{\partial I}{\partial F} = 0\,. \qquad [35]$$

This implies that the net cellular current must be constant across the distribution. However, the net cellular current exiting the region enclosed by 0 and any finite fluorescence value is 0. Since no cells can exit the distribution at $F$=0, this implies that the net cellular current must vanish everywhere:

$$I(F) = const = 0\,. \qquad [36]$$

We also note the following:

$$\begin{aligned}\Delta I(F, F+\Delta F) &= \left[I_{L\to H}(F+\Delta F) - I_{L\to H}(F)\right] - \left[I_{H\to L}(F+\Delta F) - I_{H\to L}(F)\right] \\ &\approx \left(\frac{\partial I_{L\to H}}{\partial F} - \frac{\partial I_{H\to L}}{\partial F}\right)\Delta F = 0 \Rightarrow \frac{\partial I_{L\to H}}{\partial F} = \frac{\partial I_{H\to L}}{\partial F}\end{aligned}\,, \qquad [37]$$

From where we obtain a relationship for the directional currents

$$I_{L\to H}(F) = I_{H\to L}(F) \qquad [38]$$

In summary, the up- and downward directional currents should cancel and the net cellular current should be 0 across any preset threshold or within any preset region of a stationary distribution in the absence of instantaneous fitness changes. This is somewhat equivalent to non-interacting charged particles in thermal motion that randomly cross over a preset section or volume in a cable. While separate currents may exist in both directions, for large enough cross-sections, in the absence of power sources they cancel each other out and the net current is 0. In electricity, the continuity equation has similar integral and differential forms as the cellular current above. Yet, a major difference between the cable and the cell population is that the latter is in constant growth. This is equivalent to electromotive forces pumping charges uniformly into the cable at every point along its length. Since the rate of charge accumulation is uniform along the cable, it will not contribute to the net current across any section of the distribution.

The situation changes, however, when the sources pump charge into the cable at varying rates along its length. In this case, a net current appears that is determined by the difference between the rates of charge accumulation at various points along the cable. Likewise, fluorescence-dependent instantaneous fitness changes (due to rtTA activation, for example) can have serious implications for the cellular current. Specifically, when local fitness is greater than overall fitness, $g(F) > g_T$, the extra cells produced per unit time must leave the region to reduce the rate of cell number increase to $g_T[\partial N(F,t)/\partial F]\Delta F$ . Therefore, regions of higher fitness within a distribution will act as electromotive forces that constantly pump cells into the distribution. Consequently, at stationary state the continuity equation should equate the net cellular current $I$, exiting a region enclosed by fluorescence values $F_1 < F_2$ with "the cell number increase expected from the local fitness" minus "the actual cell number increase inferred from overall fitness":

$$\Delta I(F_1, F_2) = \langle g(F_1, F_2) \rangle N(F_1, F_2) - g_T N(F_1, F_2) \qquad [39]$$

Taking $F_1 = F$ and $F_2 = F + \Delta F$, after approximating the number of cells in the region by $\Delta F[\partial N(F,t)/\partial F]$, the continuity equation becomes:

$$\Delta I(F, F + \Delta F) = \frac{\partial I}{\partial F}\Delta F = g(F)\frac{\partial N}{\partial F}\Delta F - g_T \frac{\partial N}{\partial F}\Delta F = [g(F) - g_T]\frac{\partial N}{\partial F}\Delta F \qquad [40]$$

This can be written in the differential form

$$\frac{\partial I}{\partial F} = [g(F) - g_T]\frac{\partial N}{\partial F} \; . \qquad [41]$$

### *6.1. Calculating the downward cellular current for the PF gene circuit*

The change in cell number from 0 to $F$ fluorescence, $N(0,F)$, can be attributed to two factors: cell division and biochemical kinetics. Since no cells transition from positive fluorescence to negative fluorescence, this can be expressed by the relationship

$$\frac{dN}{dt} = \frac{\partial N}{\partial t} - \frac{\partial N}{\partial F}\frac{\partial F}{\partial t}$$

The first term on the right hand side (RHS) is the cell division rate, $\partial N(0,F)/\partial t = g(0,F)N(0,F)$. The second term is the net cellular current at fluorescence $F$ (recall that cellular current is 0 across $F=0$, from positive to negative fluorescence).

The net cellular current is the product of two terms. The first of these terms refers to the density of cells per unit fluorescence, $\partial N(F,t)/\partial F$. Practically, this is estimated from flow cytometry data as the number of cells in a fluorescence bin divided by the range of fluorescence (bin width). The second term, $\partial F/\partial t$, refers to biochemical dynamics, and can be written as

$$\frac{\partial F}{\partial t} = \beta(F) - \delta(F) \, .$$

where $\beta$ and $\delta$ are birth and death rates, respectively, in the abstract sense – meaning that death can occur through dilution (not just active degradation).

For stable proteins, such as yEGFP::ZeoR, the primary cause of downward cellular current within stationary fluorescence distributions is dilution due to cell growth. For example, considering the PF gene circuit, the number of yEGFP::ZeoR proteins is practically unaffected by active degradation. Rather, yEGFP::ZeoR protein concentration dilutes out due to cell growth, which is practically the only mechanism lowering the fluorescence concentration *F*. Based on the definition of the cellular current [29], assuming that cell division rate is also the rate at which cells grow and dilute proteins,

$\frac{\partial F}{\partial t} = \beta(F) - Fg(F)$, which implies that: $\frac{dN(0,F)}{dt} = g(0,F)N(0,F) - \frac{\partial N(F,t)}{\partial F}\left[\beta(F) - Fg(F)\right]$,

and

$$I_{H\to L}(F) = \frac{\partial N(F,t)}{\partial F} Fg(F). \qquad [42]$$

### 6.2. *Calculating the upward cellular current for the PF gene circuit*

The upward cellular current is subject to many unknown or difficult-to-model factors. Translation of yEGFP::ZeoR molecules is affected by ribosomal reactions, polymerase reactions, rtTA binding to DNA, and other confounding mechanisms. Thus the gradient of the upward cellular current can be obtained indirectly by rearranging [31,41] to give:

$$\frac{\partial I_{L\to H}(F)}{\partial F} = \frac{\partial I_{H\to L}(F)}{\partial F} + \left[g(F) - g_T\right]\frac{\partial N(F)}{\partial F}. \qquad [43]$$

By integration we obtain the upward cellular current as

$$I_{L\to H}(\theta) = I_{H\to L}(\theta) + N_T \int_0^{\theta} \left[g(F) - g_T\right] p(F)\, dF. \qquad [44]$$

### 6.3. *Estimating the cellular memory based on the cellular current*

We estimated the fraction of cells (*r*)ising out of the (*L*)ow state as

$$r = \frac{I_{L\to H}(\theta)}{N_L}, \qquad [45]$$

while the fraction of cells (*f*)alling out of the (*H*)igh state is

$$f = \frac{I_{H\to L}(\theta)}{N_H}. \qquad [46]$$

Since $N_T$ is present in all of the cellular current terms, the rise and fall rates may be calculated by the fraction of cells in the low ($N_L$/ $N_T$) and high ($N_H$/ $N_T$) expressor states without explicitly measuring $N_L$, $N_H$, or $N_T$.

Memory is defined as inversely proportional to the rate at which cells leave a state,

$$\tau_L = r^{-1} \ln(2) \quad [47]$$

$$\tau_H = f^{-1} \ln(2), \quad [48]$$

with the ln(2) term implying a “half life” form of cellular memory.

### *6.4. Estimating the error from cellular current*

To estimate the cellular current, we chose boundaries between low and high expressor states at the minima between the two fluorescence peaks. Since the number of cells collected in the chosen flow cytometry bins can be quite low (often empty), the resolution of $p(F)$ may be poor. The rising rate ($r$) was not severely affected by this, since it was determined from the sum of the small downward (high to low) current *and* an integral over a large number of cells. To quantify the resolution (the smallest observable difference in $f$) we calculated $f$ assuming that only 1 cell had been observed within a flow cytometry bin at the fluorescence boundary. We observed a resolution of ~0.0208 /h.

To increase the resolution of $f$ estimation, we smoothed the distributions until the minima between peaks became non-zero. The highest level of smoothing used was $s$=80, with a correspondig resolution of ~$2.6\times 10^{-4}$ /h. However, this increased resolution came at a cost of overestimating the probability of cells being in the minimal bin, which in turn resulted in overestimates of $f$ and underestimates of the high expressor memory. Thus, cellular current provides a lower bound of the actual cellular memory for the high expression state.

### *6.5. Cellular memory estimations for different gene circuits and inducer concentrations*

We applied the cellular current method to estimate the cellular memory of yeast cells carrying the PF gene circuit at [ATc]=10 ng/ml (Figure 4E) and [ATc]=5 ng/ml (Figure S3E), as well as for yeast cells carrying the NR gene circuit [9] at [ATc]=80 ng/ml (Figure S3F).

PF cells grown in 5 ng/ml ATc were sorted into high and low yEGFP:ZeoR expressors. Memory fit using the 2 state model was estimated at $\tau_L$=40 h and $\tau_H$>$10^{13}$ h. The cellular current model estimated memory at $\tau_L$=43 h and $\tau_H$>218 h. NR cells grown at 80 ng/ml ATc were similarly sorted, and had memory estimates from the 2 state model of $\tau_L$=11 h and $\tau_H$=12 h. The cellular current estimates were $\tau_L$=14 h and $\tau_H$=8 h.

## 7. Fitness measurements

### *7.1. Measuring the resuspension period for keeping PF cells in the exponential growth*

To determine the optimal timing for periodical cell resuspension, with the aim of keeping cells in the exponential growth phase, we started cultures of PF cells ($0.05 \times 10^6$ / ml / 1ml of total

volume) in the fresh medium supplemented with 10 ng/ml of ATc (without Zeocin) and took cell count measurements using a NexCelom CelloMeter every 2-3 hours over a time period of 24 hours (Figure S7A). We found that the initial lag phase lasted approximately 5h, followed by the exponential growth phase up to approximately 18h, after which cell population growth was reduced. Based on these data, the duration of exponential growth phase for PF cells in galactose-containing media in the absence of Zeocin and ATc was estimated to be around 12 hours.

### *7.2. Fitness measurements of PF cells maintained in exponential growth*

In experiments where fitness was measured in different conditions over the period of several days, cell cultures were started from fresh colonies, and the media were supplemented with appropriate concentrations of ATc where applicable. The cells were resuspended every 12h for at least 48 hours before applying Zeocin treatment, to keep them in exponential growth and to stabilize their growth rates and yEGFP::ZeoR expression distributions. The cultures were started with cell concentrations of 0.4-1 x $10^6$ / ml (depending on ATc and Zeocin concentration, 1 ml of total volume) and were counted and resuspended in fresh media with identical composition every 12h to keep them in exponential growth. Fitness for cells with no ATc induction was measured from earlier time points, since uninduced cells had little protection from Zeocin, and were potentially under strong evolutionary pressure. Fitness for cell populations with ATc induction was estimated over the last 6 time points to minimize the transient effects immediately after exposure to Zeocin. Growth rates were obtained by linear fits to the log transformed cell counts (Figure S7B-D, Table S3).

## 8. Switching rate measurements based on the two state population-dynamics model

A previously defined two-state population-dynamics model [24,25], with gene expression states corresponding to high and low yEGFP fluorescence, was used to estimate the switching rates of cells based on sorting experiments:

$$\begin{aligned} \dot{N}_L &= -rN_L + fN_H + g_L N_L \\ \dot{N}_H &= rN_L - fN_H + g_H N_H \end{aligned} . \qquad [49]$$

where $g_L$ corresponds to the growth rate of low expressors grown, while $g_H$ corresponds to the growth rate of high expressors. Furthermore, cells in the low ($L$) state could switch ("rise") into the high ($H$) state with rate $r$, while cells in the $H$ state could switch ("fall") into the $L$ state with rate $f$. Initially, we assumed equal growth rates, $g_L = g_H = g$, but later revised the model to account for the growth retardation due to the "squelching effect" of rtTA [3,4]. Solving this system resulted in an analytical expression for the subpopulation ratio of low expressors to high expressors over time, $R(t)=N_L(t)/N_H(t)$, which was fit to the experimentally measured subpopulation ratio, as described below.

### *8.1. Estimating the non-genetic memory of high and low expressing cells by sorting bimodal cell populations*

Bimodal PF cell populations were separated by FACS into low and high expressor subpopulations and then monitored over several days together with the corresponding unsorted

PF cell populations as a control. Cells were resuspended every 12 hours and assessed by flow cytometry at different time points (see Figure S8).

The subpopulation log-ratio, $\log_{10}[N_L(t)/N_H(t)]$ obtained from the population-dynamic model was used to represent the prevalence of $L$ and $H$ cells, since the log-ratio can be represented as the difference of two log-transformed values. We assumed that unsorted cells were stationary (see Figure S9A) so that any differences in their distributions were due to systematic errors. Thus, the log-transformed subpopulation ratio for the unsorted population was used to estimate the systematic variation at each time point, defined by the equation

$$\log_{10}\left(R_{U,i}\right) = \overline{\mu_U} + \varepsilon_i \,, \qquad [50]$$

where $\overline{\mu_U}$ is the stationary mean of log-ratios, $\varepsilon_i$ is the experimental variation at the $i^{th}$ time point, and $R_{u,i}$ is the subpopulation ratio measured at the $i^{th}$ time point for unsorted cells ($U$). The mean log-ratio is estimated as

$$\overline{\mu_U} = \frac{1}{n}\sum_{i=1}^{n}\log_{10} R_{U,i} \,. \qquad [51]$$

If the experimental error is systematic, with identical effects on both sorted and unsorted cells, then the mean number of cells at the $i^{\text{th}}$ time point is given by

$$\log_{10} R_{P,i} = \mu_{P,i} + \varepsilon_i \,, \qquad [52]$$

where $R_{P,i}$ is the subpopulation ratio at the $i^{\text{th}}$ time point, and $\mu_{P,i}$ is the average log-ratio at the $i^{\text{th}}$ time point in the cell population of type $P$ (i.e. low-sorted, high-sorted or unsorted populations). Rearranging [52] and substituting $\varepsilon_i$ from [50] gives the "normalized" mean subpopulation ratio,

$$\mu_{P,i} = \log_{10} R_{P,i} - \log_{10} R_{U,i} + \overline{\mu_U} \,. \qquad [53]$$

The theoretical estimates of $R=N_L/N_H$ from [23] were fit simultaneously to all three time courses of the subpopulation ratios measured in the 3 cell population types (unsorted, low-sorted, and high-sorted taken from a single experiment). The 3 subpopulation ratio measurements were first normalized according to [53]. The only parameter within the model allowed to change was the starting subpopulation ratio, obtained from the experimental data. The error function used to estimate how appropriate the parameters were to model the data was defined as

$$\begin{aligned} E_P &= \sum_i \left[\log_{10}\left(\hat{\mu}_{P,i}\right) - \log_{10}\left(\mu_{P,i}\right)\right]^2 \\ E_{Total} &= E_L + E_H + E_U \end{aligned} \,, \qquad [54]$$

where $\hat{\mu}$ is given by the log-transformed analytical subpopulation ratio from [53], $E_P$ is the error associated with the cell population of type $P$, and the total error $E_{Total}$ (which was minimized to obtain switching rates) is defined as the sum of errors for the low, high, and unsorted populations. This had the practical effect of attributing 1/3 of the error weighting to the unsorted

population, forcing all parameters to values that ensure the asymptotic convergence of all solutions to the subpopulation ratios of unsorted population.

The parameters $r$, $f$ were estimated individually from triplicate experiments, and averaged. Starting $R(0)$ values were taken as the geometric mean of the starting ratios of modes, to ensure that populations with predominately high expressors are weighted equally to populations with predominantly low expressors. Estimated parameters included the switching rates $r$ and $f$, as well as the starting ratios $R_L(0)$ and $R_H(0)$ of (L)ow to (H)igh expressors at the first time point. Growth was estimated separately for unsorted populations based on cell counts using a NexCelom Cellometer. We note that when estimating the ratios of (L)ow to (H)igh expressors, we first assumed equal growth rates for the mathematical model of growth).
Minimizing [54] using Matlab's fminsearch algorithm, while constraining the growth rates of high and low expressors to be equal, we obtained switching rates of $r \approx 1.25\times10^{-2}$ h$^{-1}$; $f = 7.1\times10^{-3}$ h$^{-1}$ for PF cells grown at 10 ng/ml ATc. The fit appeared to consistently underestimate $r$ while overestimating $f$ (Figure S9B).

### *8.2. Using purified subpopulations of high-expressing PF cells to estimate cellular memory*

We took advantage of the strong non-genetic memory of the high expression state in PF cells and established a method to obtain purified populations of high expressing PF cells (>99% purity) following the seminal paper on lac operon bistability by Novick and Weiner [26]. Briefly, PF cells were first pre-induced to the bimodal expression state using ATc = 10 ng/ml (Figure S10A). Then we performed serial dilutions and prepared 80 tubes with a concentration of approximately 1 cell per 10 tubes (0.1 cell/ml). Cultures were grown from these isolated cells, periodically resuspending them in fresh medium with the same ATc = 10 ng/ml for 4 days. Since a period of 4 days is shorter than the memory of high expression, but higher than the memory of low expression, the expectation was that subsequent cultures started from a single low expressor will have bimodal distribution while cultures started from a single high expressor should predominantly consist of high expressors. As expected, by day 4 we obtained two different types of cell populations, some of which indeed consisted more than 99% of high-expressing cells (Figure S10B).

By periodically resuspending these cultures into fresh medium and monitoring their gene expression pattern by flow cytometry, we also estimated the non-genetic memory of high expressors as in the sorting experiments described in the main text. As expected, the populations that initially consisted almost entirely of high expressing cells eventually relaxed to the bimodal distribution over several days (Figure S10C). These results confirmed that the non-genetic memory of high expressors greatly exceeded the memory of low expression (see the main text).

## 9. Ideal phenotypic switching rates

### *9.1. Mutual dependence of switching rates and fitness affects optimal survival strategies*

Cells unable to sense environmental changes can maximize their fitness by stochastically transitioning between different phenotypes [27,28]. However, these switching rates must be reasonably in tune with the environment to have a strong benefit. Kussell and Leibler found that cells whose fitness was unaffected by switching would have ideal switching rates proportional to the rate at which the environment switched, and inversely proportional to the time spent in an

environment [28]. For our two-state system, the corresponding ideal memories based on the Kussell-Leibler model are predicted to be

$$\begin{aligned} &\tau_H(\text{optimal}) = T^2_{toxic}(\text{optimal}) \\ &-\text{and}- \qquad , \\ &\tau_L(\text{optimal}) = T^2_{normal}(\text{optimal}) \end{aligned} \qquad [55]$$

where $\tau_L$ and $\tau_H$ are the memories of the low and high state cells, and $T_{toxic}$ and $T_{normal}$ are the average duration of the toxic and normal environments, respectively.

Does this prediction change when switching rates and cellular fitness are mutually interdependent? In order to answer this question, we created a set of functions that reasonably approximated experimental rates at 0 and 2 mg/ml Zeocin (see Figure S11A and B). These rates followed experimental rates to maintain biological plausibility, but were not meant to be predictive. We then simulated cells switching in either a periodically oscillating or a randomly fluctuating environment. The first environmental switching rates were predicted to have optimal memories at 10 ng/ml ATc (see Figure S11C) and 100 ng/ml ATc(see Figure S11D). Next, we simulated environmental switching rates with long intervals in the toxic environment and short intervals in the normal environment (cells would be predicted to be most fit incubated in saturating ATc, see Figure S11E). Finally, cells were simulated in a purely toxic, constant environment (see Figure S11F). In contrast to the Kussell-Leibler predictions, these simulations suggest that cells with a fitness cost to the rate of switching will benefit more from limiting their switching rate than from having switching rates that mirror environmental rates.

### *9.2. The "sweet spot" occurs when switching rates are less than growth rates*

We next asked whether the observed sweet spot was due to the ratio of switching rates, or whether memory was important. Using we scaled the switching rates by a constant *k* (*r'=kr*, *f'=kf*). As the switching rates became comparable to growth rates (at k=10), the sweet spot was dampened and disappeared at high switching rates (see Figure S11G).

## 10. Gillespie simulations of cell populations maintained in exponential growth

Gillespie simulations of PF cells were simulated using Matlab's *ssa* (stochastic simulation algorithm) solver from the SimBiology toolkit. In addition to simulating chemical kinetics, cells divided at rates determined by rtTA toxicity. The time to cell division was simulated by a constitutively expressed "counter" protein driving cell cycle forward, in such a way that the mean division time mirrored those from experimental measurements, with a division time CV of ~15%. To obtain a CV of ~15% for completely uninduced cells, cell division was defined to occur when the number of counter proteins reached 89, with cells on average starting with 45 counter molecules. ATc-bound rtTA was assumed to repress cellular transcription (squelching effect) causing a fitness reduction. This was simulated by having rtTA bind to a constitutive protein responsible for driving the cell cycle counter (see Figure S12).

Upon division free floating molecules and cell volumes were reapportioned between two cells according to a binomial distribution with equal probabilities (p=0.5) [29,30]. This represents a slightly more complicated form of the previously defined growth "electromotive force analogue" ([41]), which is given as

$$\frac{\partial N_{Growth}(F)}{\partial t} = \int_{-\infty}^{\infty} \rho(F-\omega) g(\omega) n(\omega) d\omega \,. \qquad [56]$$

where $\rho$ is the probability of reapportionment. When $F \neq \omega$, the reapportionment is unequal. The cellular current equation was derived with $\rho$ defined as a delta function.

Since cell division can imply an exponential increase in the number of simulations required, cells were made to die at rates comparable to their growth rates. 5000 cell populations starting from a single uninduced cell were simulated over 96 hours, with frequencies obtained by sampling yEGFP::ZeoR protein levels from the last 48 hours of cell simulations.

PF cells were simulated with rates consistent with previous simulations of TetR systems [9], taking into account the differences between rtTA and TetR, and comprise lower ATc binding rates, lower DNA binding affinity, and a degradation half time of 20 minutes [31,32].

Stochastic models were developed in Dizzy [33] (see SI Dizzy code), and then converted into a Matlab SimBiology model for cell population-level simulations.

## 11. Tracking cells from micoscopic images obtained in a microfluidic device

Brightfield and fluorescence images of dividing cells were obtained over 48 hours. We used custom tracking algorithms to track cells over 30 hours and obtain confirmatory statistics (see Figure S12C). We estimated the rise (*r*) and fall (*f*) rates by counting the number of cells that crossed a predefined threshold, divided by the time over which these crossings were observed and the number of cells above and below the threshold immediately before counting. Using a threshold boundary of 2000 arbitrary fluorescence units, we found that the ratio of low to high expressors was 1.27. The estimated rise and fall rates were $r = 28.7\times10^{-3}$ $h^{-1}$ and $f = 3.9\times10^{-3}$ $h^{-1}$, representing a 7-fold difference in switching rates, although the subpopulation ratio was approximately 5:4. In conclusion, this analysis confirmed that switching from the OFF state into the ON state occurred much more frequently than the reverse transition over the time period of observation, corroborating our cellular memory estimates from the main text.

| Primer name | Sequence (5’ -> 3’) |
|---|---|
| Tetreg-AflII-f | GCGCCTTAAGGCGCCACTTCTAAATAAGCGAATTTC |
| CYC1-BamHI-r | GCGCGGATCCCCCGAATTGATCCGGTAATTTAGTG |
| T2-Down-f | AGAGTCGACAAAGTCGAGTTTCTCGATCCCTATCAGTGATAG |
| T2-Up-r | CTATCACTGATAGGGATCGAGAAACTCGACTTTGTCGACTCTC |
| Amp-AhdI-f | GCCTGACTCCCCGTCGTGTAGATAACTACG |
| HIS3-Add1-r | AAGCAATAGTAGAAAAAAACAATGATATGGTGCACTCTCAGTAC |
| HIS3-Add2-r | CTTTCGATAAGTTTTTCCCACAGCAAAGCAATAGTAGAAAAAAAC |
| HIS3-Add3-r | ACGAGAATTAAGAAAAAGTCGTCATCTTTCGATAAGTTTTTCC |
| HIS3-AfeI-r | TCCTAGCGCTCACCAAGCTCTTAAAACGAGAATTAAGAAAAAGTCG |
| Origin-PstI-f | AAAGCTGCAGCATACGATATATATACATGTGTATATATG |
| Origin-SacI-r | GCGCGAGCTCCAGCTGGCGTAATAGCGAAGAGG |
| VP16-XhoI-r | GCGCCTCGAGTTACGATCCCCCACCGTACTCG |
| Origin-AflII-r | GCGCCTTAAGCAGCTGGCGTAATAGCGAAGAGG |
| rtTA-BamHI2-f | GCGCGGATCCATGTCTAGATTAGATAAAAGTAAAG |
| FFF-Add-r | GTCGAAATCGTCAAGAGCATCTGCAGGCAACATATCCAAATCAAAGTCATCCAAG GCGTCAGCTGGACCCCCACTTTCACATTTAAGTTG |
| FFF-XhoI-r | GCGCCTCGAGTTAACCTGGCAACATATCTAAATCAAAGTCATCTAATGCGTCGGC GGGTAGCATGTCTAGGTCGAAATCGTCAAGAGCA |
| Backbone-r | CGCGTTGGCCGATTCATTAATGC |
| TRP-f | ATGTCTGTTATTAATTTCACAGGTAGTTC |
| TRP-r | CTATTTCTTAGCATTTTTGACGAAATTTG |
| Origin-middle-f | CTATTAAAGAACGTGGACTCCAACGTCAAAG |
| Second-tetO-f | AGAGTCGACAAAGTCGAGTTTCTCGATCCC |
| CYC1-r | CATACAGAGCACATGCATGC |
| GalSeqE-r | TGAATAATTCTTCACCTTTAG |
| HISSeq-r | CGGTATTTCACACCGCATAGATCCGTCGAG |
| HISEnd-f | TTCCCTCCACCAAAGGTGTTCTTATG |
| TADH-r | CTGACCTACAGGAAAGAGTTACTCAAGAATAAG |

***Table S1****. Oligonucleotides used for cloning, sequencing and yeast strain validation.*

| Parameter | PF [Eq. 3] |
|---|---|
| $a$ | 180 nM $h^{-1}$ |
| $b$ | 3 $nM^{-1}$ $h^{-1}$ |
| $C$ | 7 [ATc] $h^{-1}$ |
| $\delta$ | ln(2)/0.33 $h^{-1}$ |
| $g$ | 0.24 $h^{-1}$ |
| $h$ | 1 $h^{-1}$ |
| $l$ | 2.5 nM $h^{-1}$ |
| $n$ | 2 |
| $\theta$ | 34.4 nM |
| $\Omega$ | 1 |

***Table S2.*** *Parameters describing PF dynamics based on equation* [3].

| ATc (ng/ml) | Zeocin (mg/ml) | Fitness ($h^{-1}$) |
|---|---|---|
| 0 | 0 | 0.2374 |
| 1 | 0 | 0.2181 |
| 2.5 | 0 | 0.2174 |
| 5 | 0 | 0.2115 |
| 10 | 0 | 0.1846 |
| 20 | 0 | 0.1527 |
| 0 | 0 | 0.2573 |
| 0 | 0.25 | 0.2371 |
| 0 | 0.5 | 0.2215 |
| 0 | 1 | 0.1806 |
| 0 | 2 | 0.0522 |
| 0 | 2 | 0.0619 |
| 1 | 2 | 0.1885 |
| 2.5 | 2 | 0.1739 |
| 5 | 2 | 0.1502 |
| 10 | 2 | 0.1346 |
| 20 | 2 | 0.1247 |

***Table S3.*** *Experimentally measured growth rates of PF cell populations in various antibiotic and inducer concentrations.*

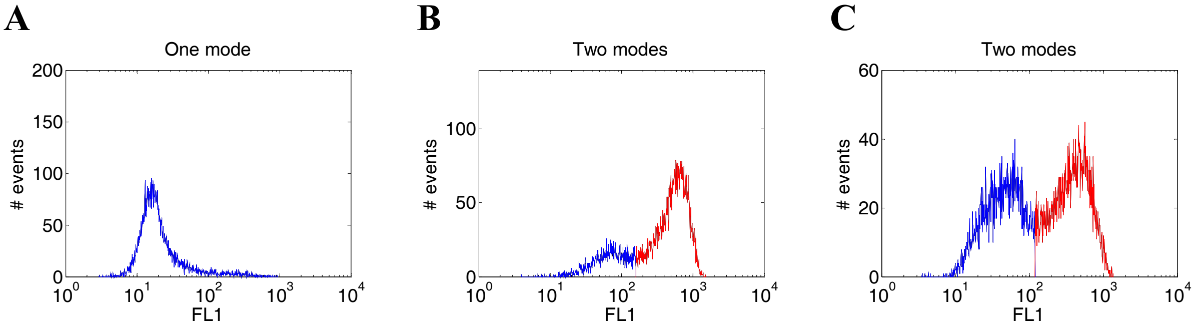


***Figure S1***. *Fluorescence histograms classified as unimodal (A) or bimodal (B & C).*

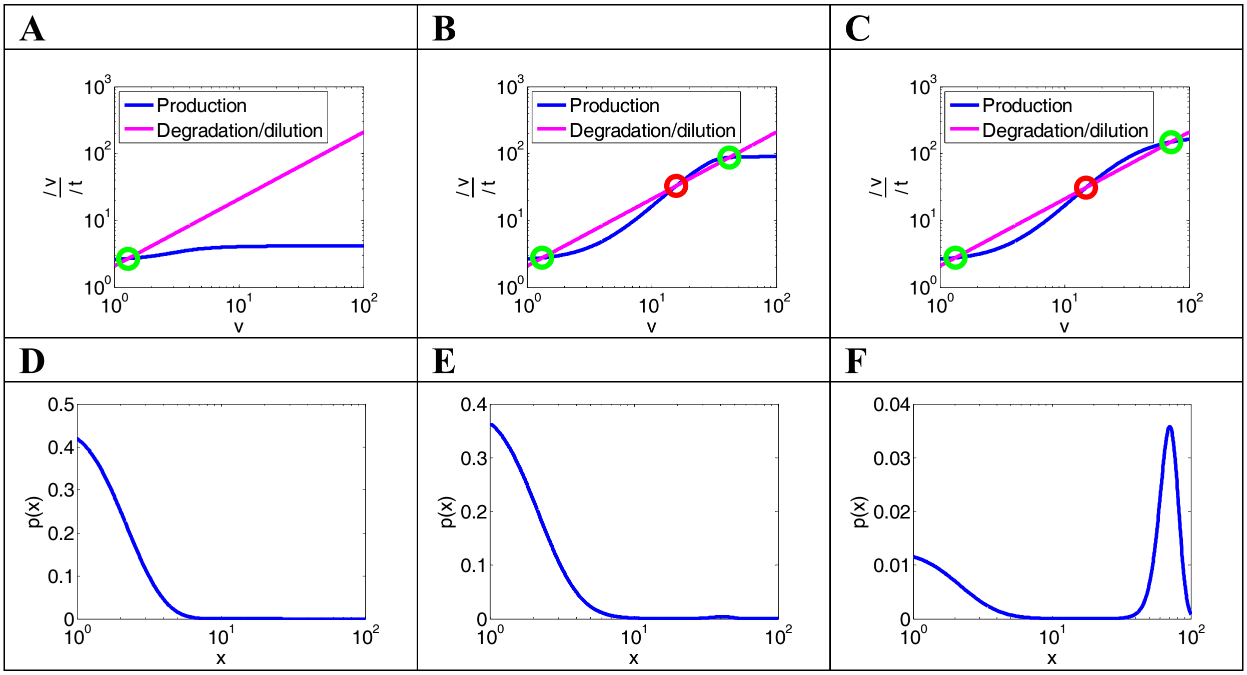


***Figure S2**. Intersection of production and degradation terms for the PF model ([5,6],parameters in table S2) at 1, 10, and 1000 ng/ml ATc are shown by circles in panels (A), (B), and (C), respectively. Green denotes stable states, red denotes the unstable states. The corresponding Fokker-Planck probability distributions are shown at 1, 10, and 1000 ng/ml ATc in panels (D), (E), and (F), respectively.*

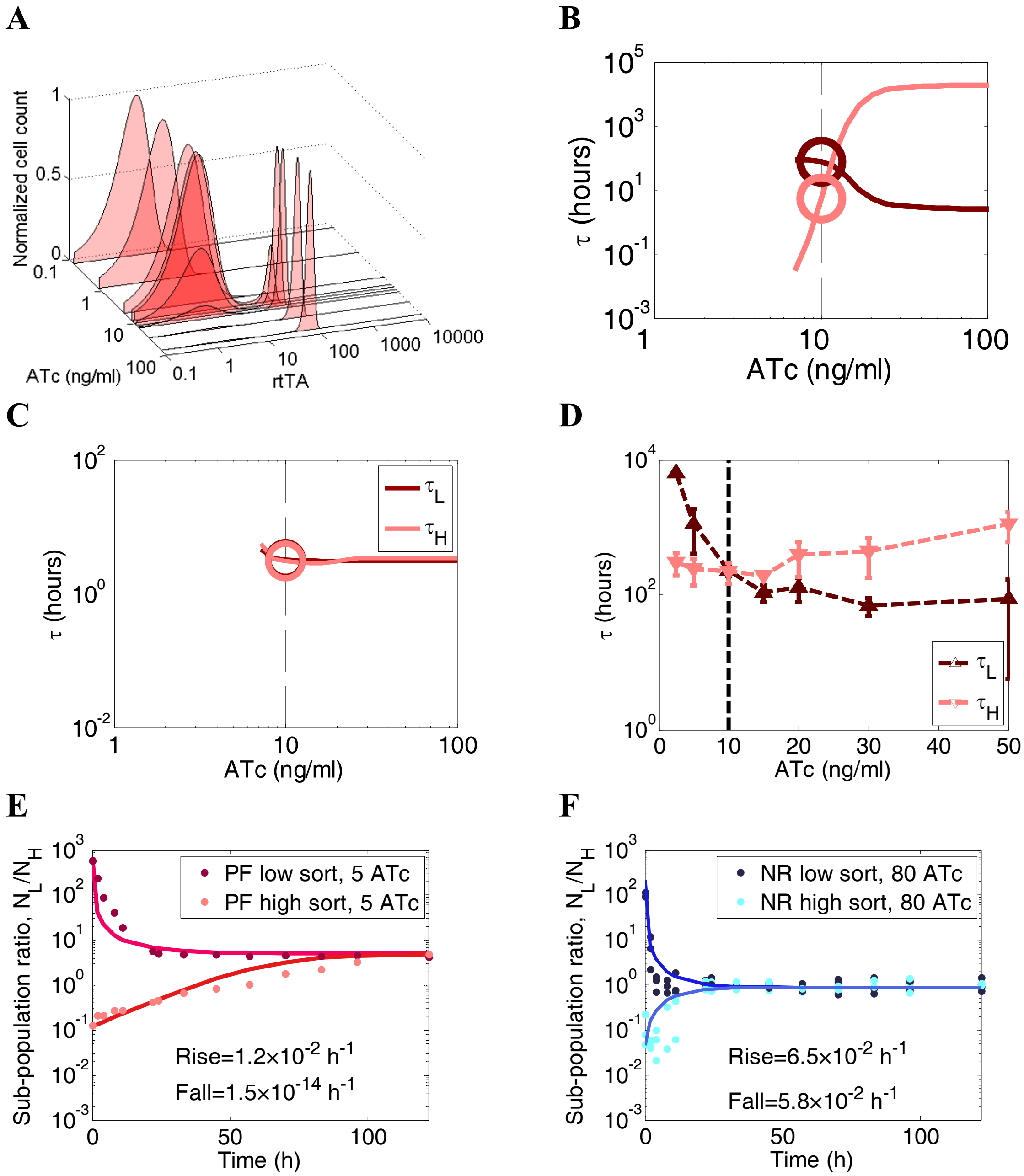


***Figure S3**. Transactivator (rtTA) expression histograms (A) and cellular memories (B) predicted using the Fokker-Planck equations and the Dynkin equation for the PF gene circuit* [9,12]. *(C) Cellular memories predicted using Kramers' escape rate method. The rates used for these calculations are listed in Table S2. (D) Estimates (based on the cellular current) of cellular memories for high and low expressor PF cells as a function of ATc, assuming cell division rates independent of ATc. Memories are calculated from the relationships $\tau_H = N_H / I_{H\to L}$ and $\tau_L = N_L / I_{L\to H}$ where $I_{H\to L} = I_{L\to H} = g\theta n(\theta)$, where $\tau$, $N$, $\theta$, and $I$ are the memory, number of cells, fluorescence threshold, and cellular currents of cells in the (H)igh or (L)ow expression states, respectively. The black dashed line shows the ATc levels where the sorting experiment was performed. Memories were also estimated by sorting for (E) PF cells at 5 ng/ml ATc and (F) NR cells at 80 ng/ml ATc.*

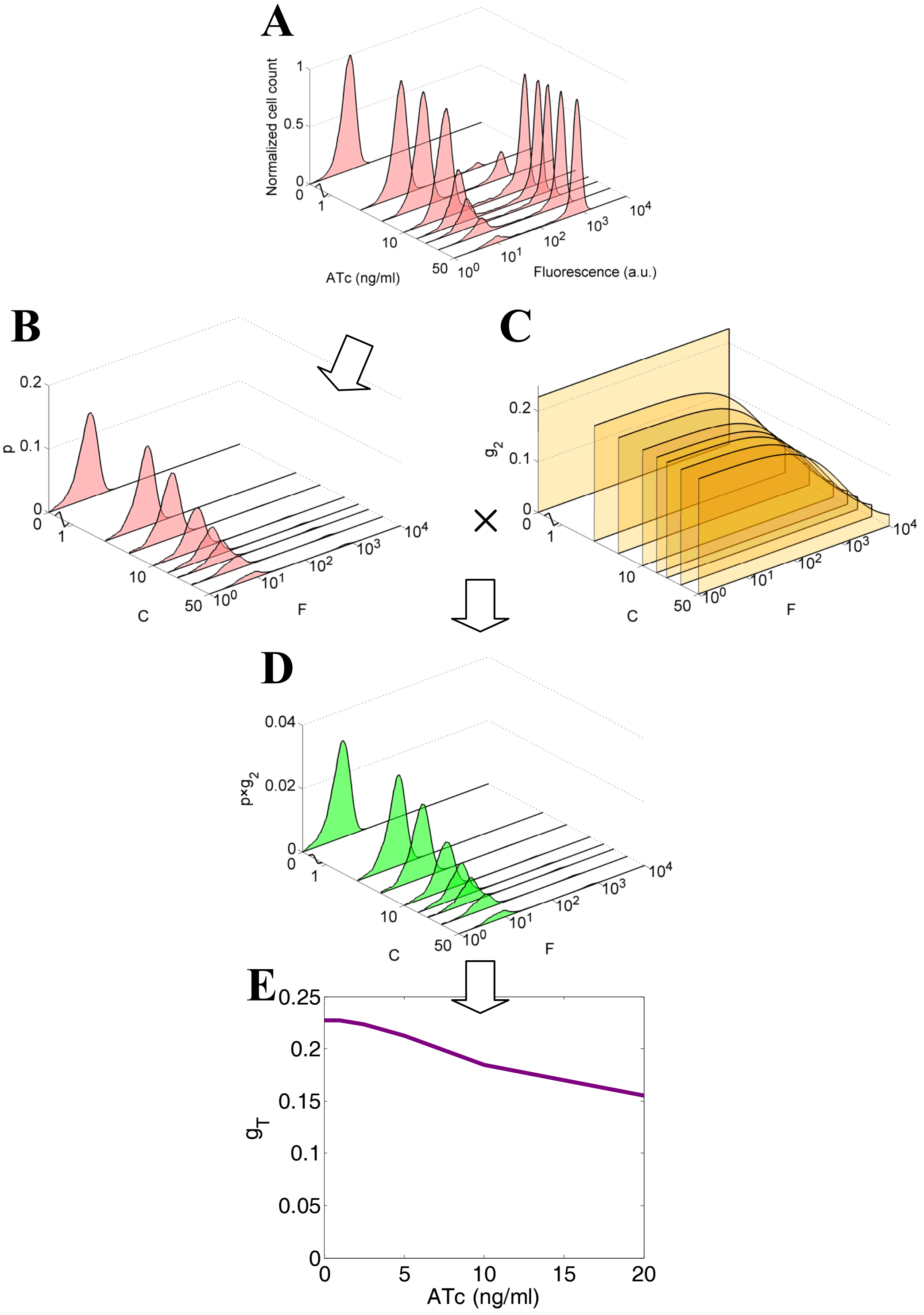


***Figure S4***. *Workflow for calculating overall cell population fitness. Logarithmically binned flow cytometry data (A) is converted to its corresponding probability density function (B). The instantaneous fitness (C) is multiplied by the probability density function (B) to obtain the "instantaneous" contribution to the overall fitness as a function of fluorescence (D). The average of* $g_2$ *is used to calculate the overall cell population fitness,* $g_T$ *(E).*

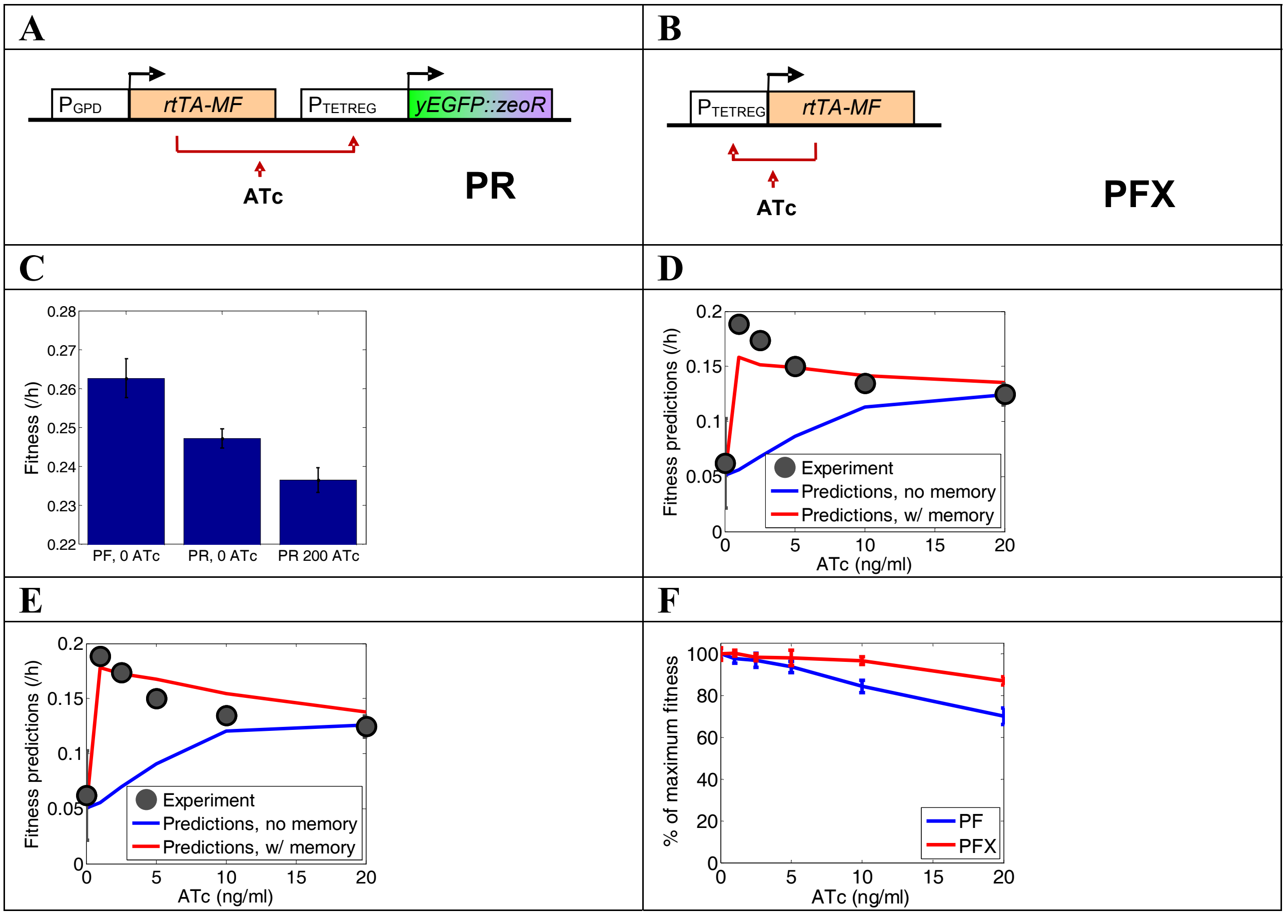


***Figure S5****. (A) The positive regulation (PR) synthetic gene circuit, consisting of the constitutively expressed rtTA-MF transactivator (from the GPD promoter) that activates the yEGFP::zeoR reporter when bound by the ATc inducer. (B) The positive feedback (PFX) synthetic gene circuit with deleted yEGFP::zeoR coding region, but otherwise identical to the PF gene circuit. (C) Overall cell population fitness decreases both with rtTA expression (fitness of PF cell populations at 0 ng/ml ATc is greater than fitness of PR cell populations at 0 ng/ml), and with rtTA activation (fitness of PR cell populations at 0 ng/ml is greater than fitness of PR cell populations at 200 ng/ml ATc). (D,E) Two alternative fitness models were tested with dependences on total rtTA concentrations (D) and active and inactive rtTA concentrations (E). Both models capture the same essential feature ("sweet spot") when memory is incorporated, but the model with dependence on active rtTA was most similar to the experimental data, consistent with experimental observations that ATc-bound rtTA has a strong contribution to fitness reduction. (F) Comparison of overall population-level fitness reduction in PF (Figure 2A) and PFX (Figure S5B) cells as a function of increasing ATc concentrations at 0 mg/ml Zeocin shows that that preventing metabolic costs of transcription and/or translation in the reporter gene is able to diminish the overall rtTA-related toxic effects in high expressor cells.*

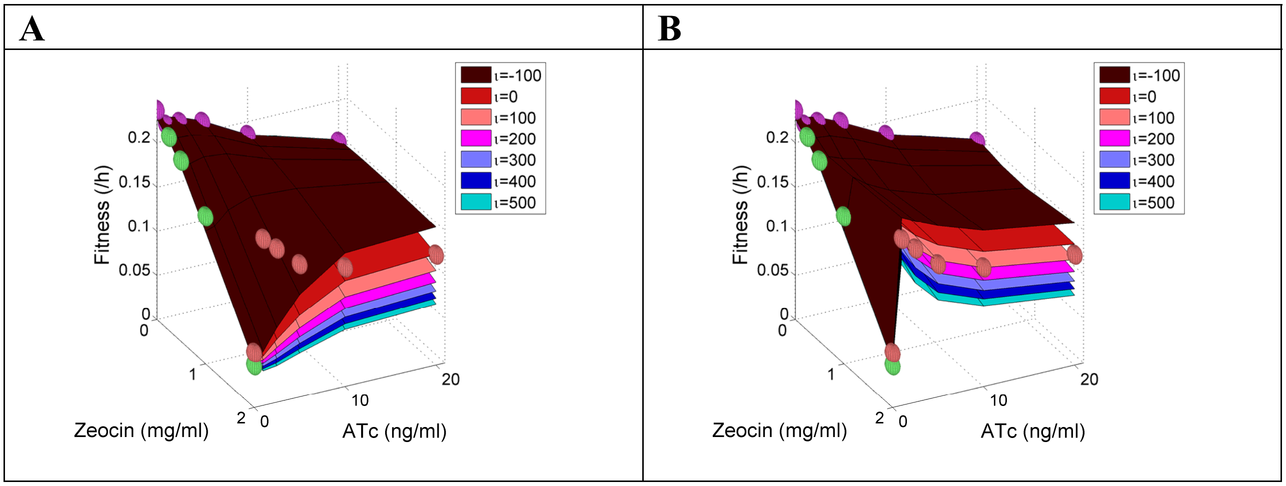


***Figure S6***. *(A) Loewe additivity does not improve memoryless predictions, regardless of synergy or antagonism. (B) Fitness prediction based on realistic memory and Loewe additivity give similar results to those based on Bliss independence. Experimental results may suggest slight synergism between rtTA toxicity and Zeocin toxicity at ι=100. Experimental data points are shown by spheres, with purple spheres corresponding to 0 Zeocin, green spheres corresponding to 0 ATc, and red spheres corresponding to2 mg/ml Zeocin.*

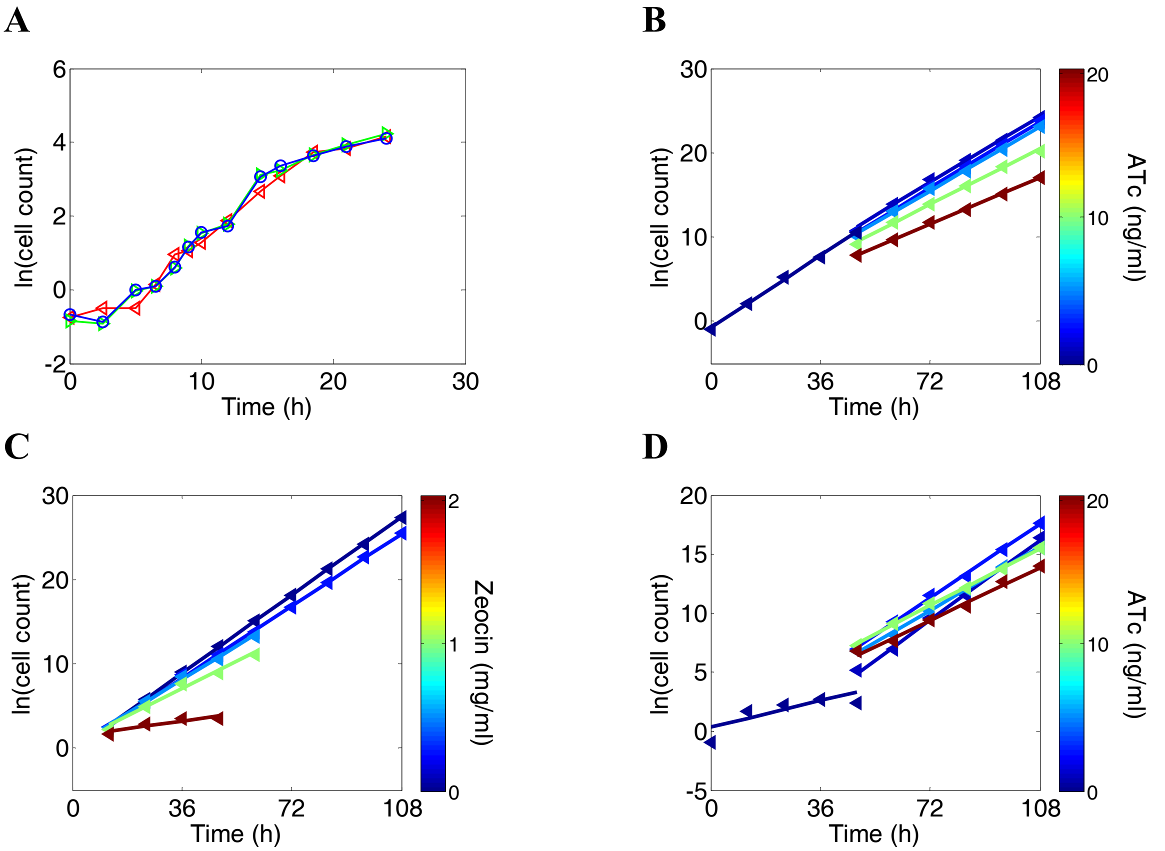


***Figure S7***. *(A) Log-transformed cell counts for PF cells at 10 ng/ml ATc and 0 mg/ml Zeocin. Log-phase lasts somewhat longer than 12 hours. (B, C, D) Growth curves of cells resuspended every 12 hours in 0 mg/ml Zeocin over a range of ATc concentrations (B), 0 ng/ml ATc over a range of Zeocin concentrations (C), and 2 mg/ml zeocin over a range of ATc concentrations (D).*

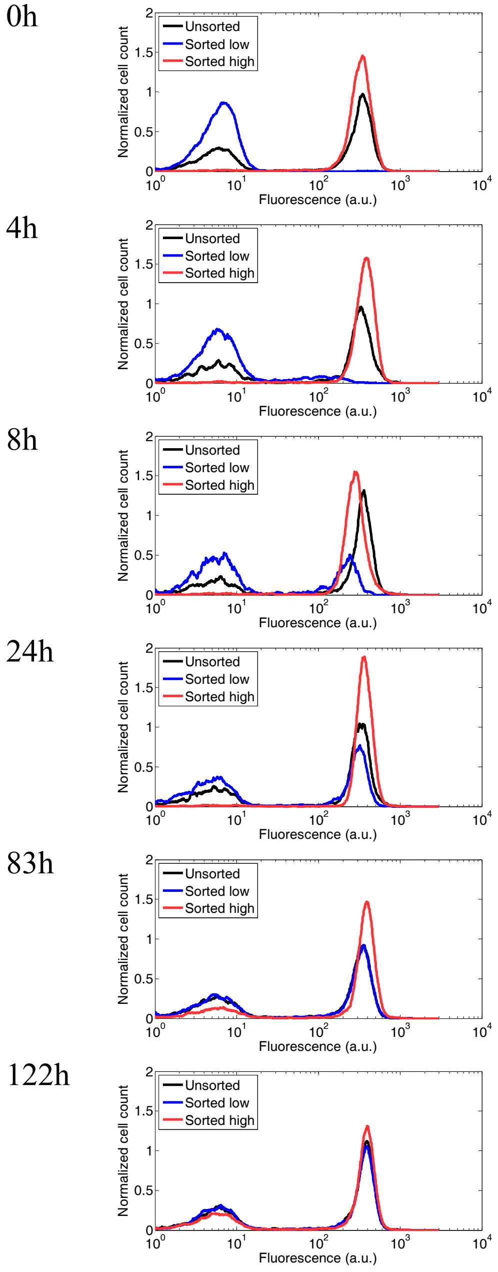


***Figure S8**. Experimental histograms for unsorted, low-sorted and high-sorted PF subpopulations grown in 10 ng/ml ATc.*

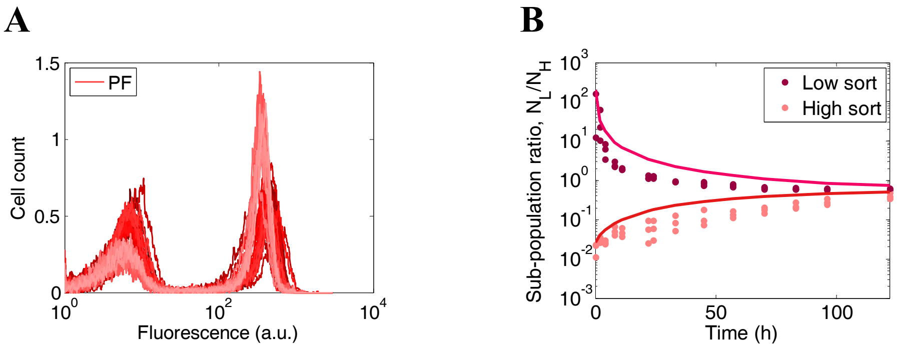


***Figure S9***. *(A) 42 unsorted PF cell populations as measured during 3 separate sorting experiments demonstrate stable steady state distributions when resuspended every 12 hours. (B) Expected relaxation to steady-state distributions based on population-dynamic models with* <u>*equal*</u> *growth rates at 10 ng/ml ATc [49]. Switching rates were estimated from [54] for PF strain with identical growth rates. The subpopulation ratio, $R= N_L/N_H$, is plotted as a function of time. Ignoring growth effects resulted in strongly distorted fits for PF sorting.*

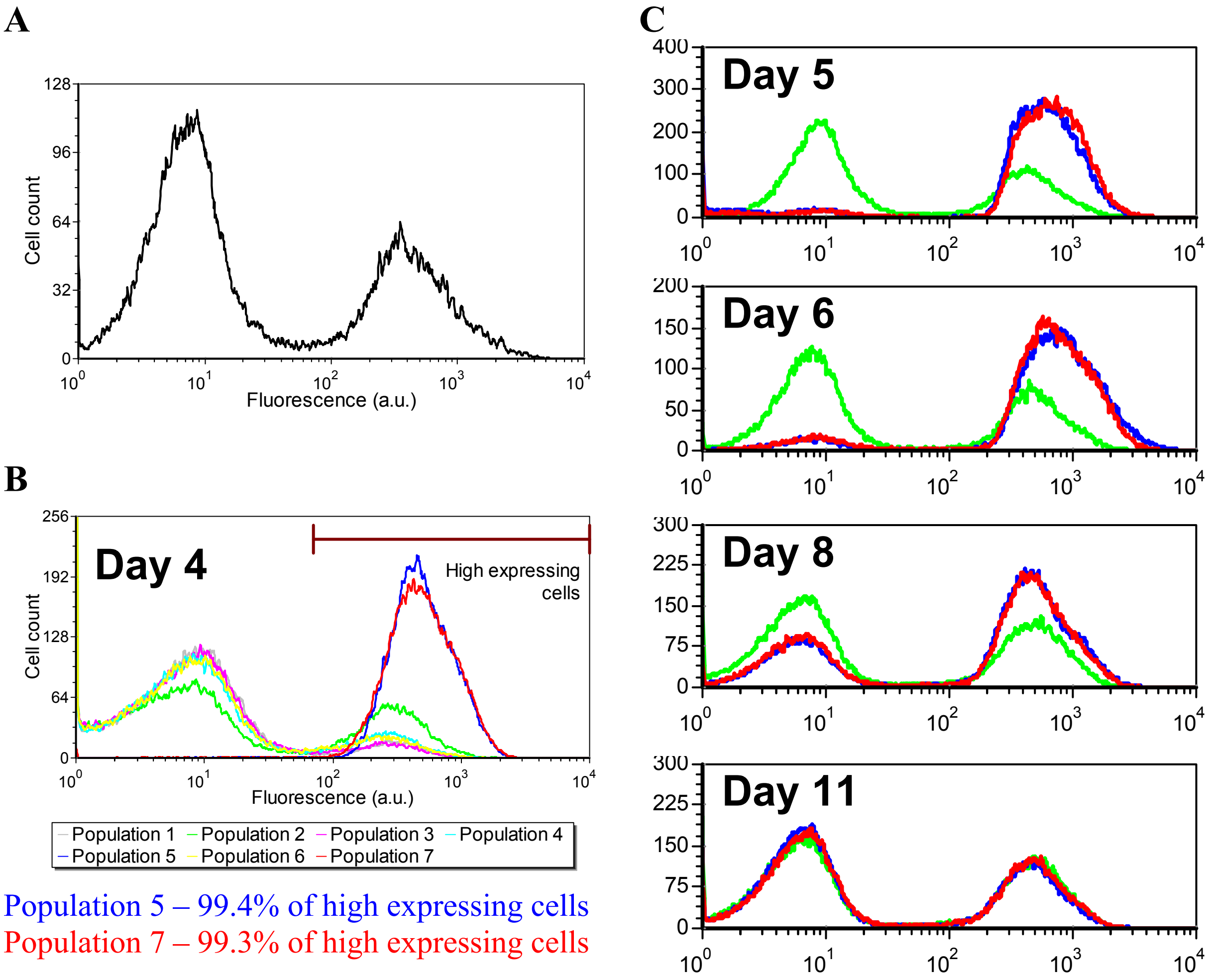


***Figure S10.*** *Serial dilution experiment for obtaining populations of PF cells grown from single cells. (A) Initial distribution of PF cells grown in 10 ng/ml ATc; (B) Populations of PF cells in different tubes after 4 days during the serial-dilution experiment. Populations 5 and 7 were highly enriched (>99%) in high expressing cells; (C) Monitoring Populations 5 and 7 (blue and red) and Population 2 (green, control bimodal population) demonstrated the strong memory of high expression, as long time was needed for the predominantly high expressing subpopulation switch to the bimodal state.*

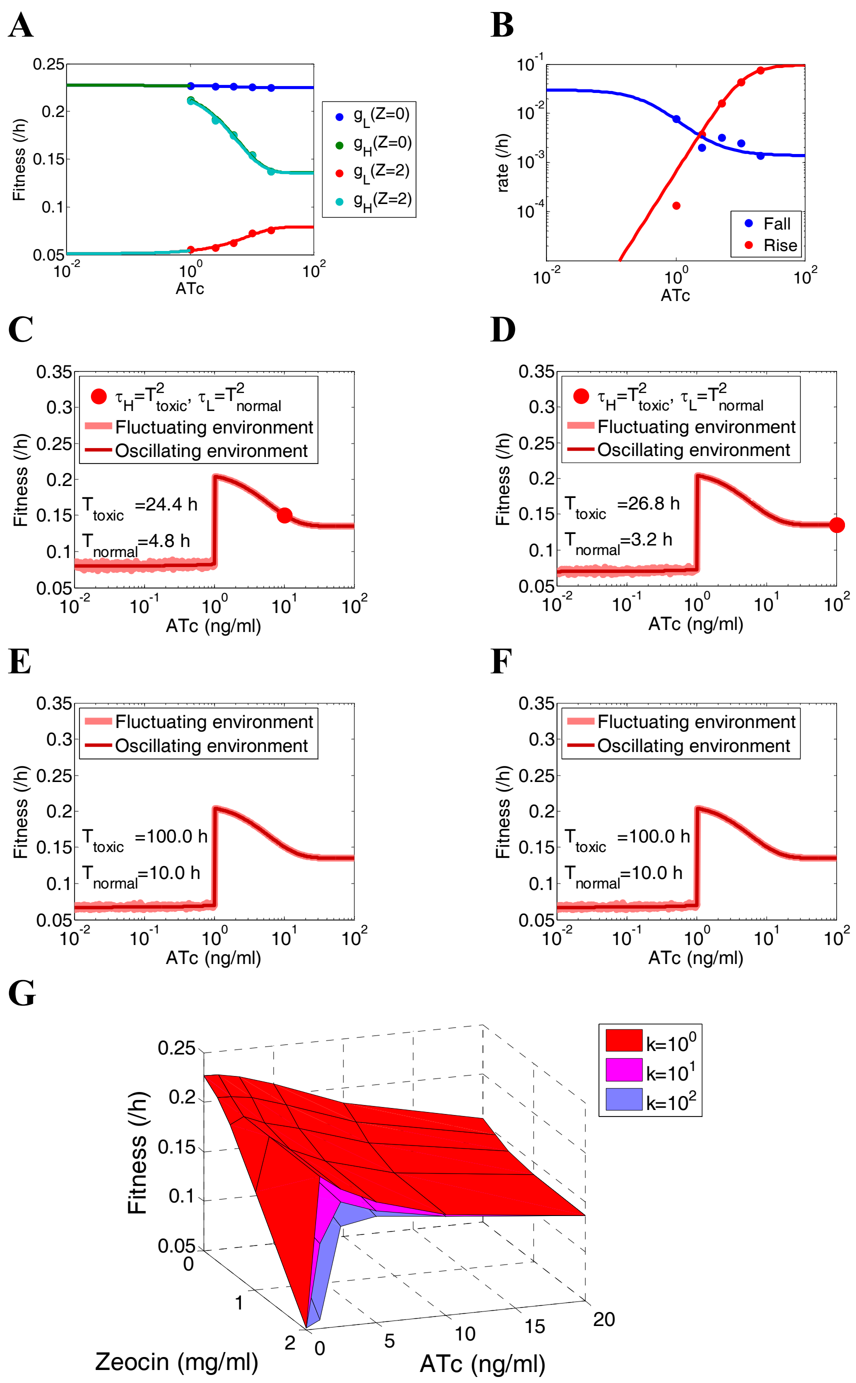


***Figure S11***. *Heuristic functions were fit to experimental fitness data at 0 and 2 mg/ml Zeocin (A) as well as switching rates (B). These fit functions were used to simulate cell growth when the environment switched between 0 and 2 mg/ml Zeocin. The red circles correspond to simulated environments with optimal phenotypic switching rates at 10 ng/ml ATc(C), 100 ng/ml ATc (D), long-lasting toxic environments (E), and a constant toxic environment (F). Predicted ideal switching rates do not agree with simulated ideal switching rates. (G) Actual switching rates scaled by the factor k=1, 10, 100. As switching rates reach parity with growth rates (k=10), the sharp peak defining the sweet spot disappears.*

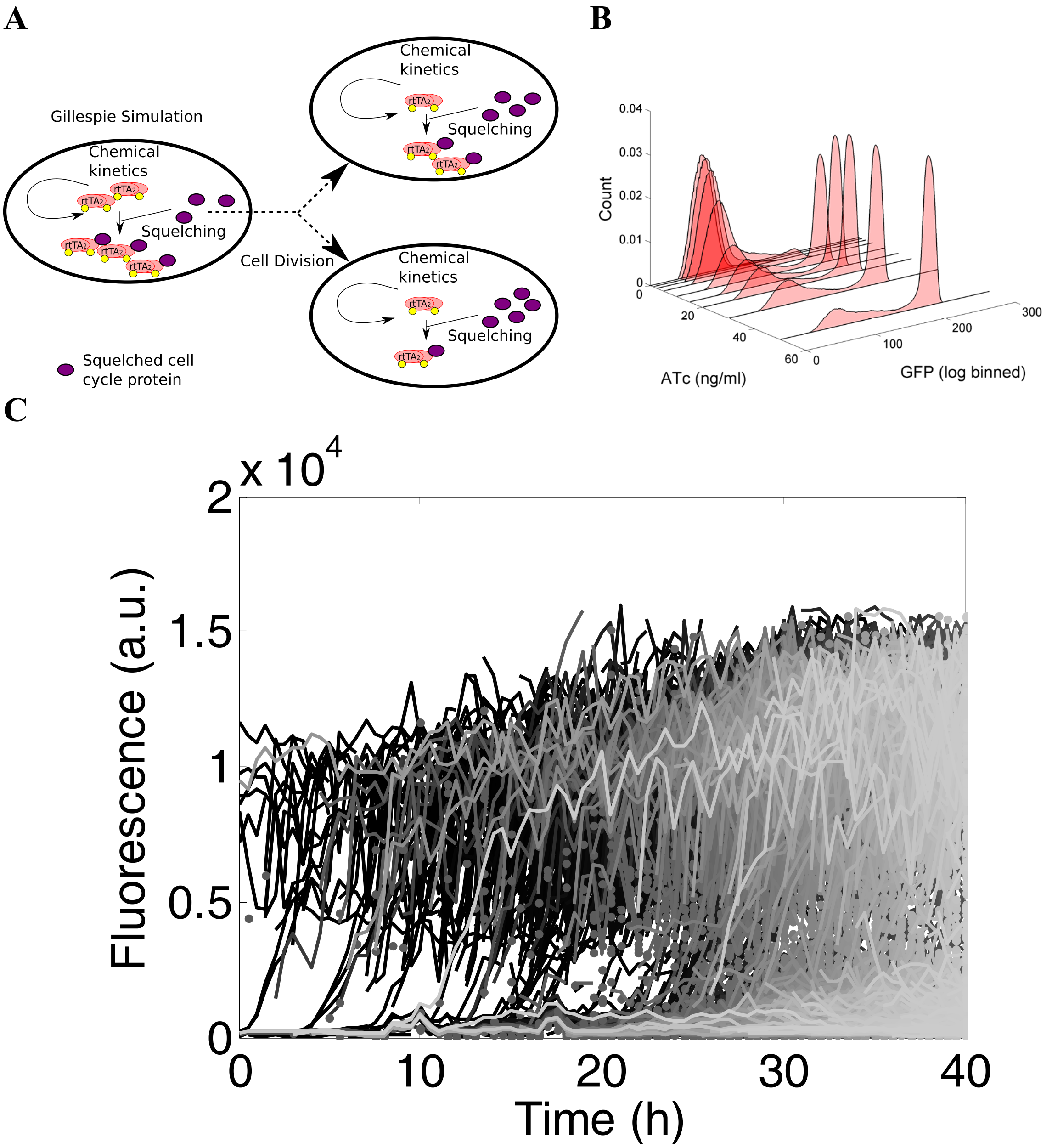


***Figure S12***. *Schematic representation of stochastic simulations of cell population growth and live cell imaging. (A) Intracellular chemical kinetics are simulated using the Gillespie algorithm. Cell cycle proteins drive "counter" molecules that initiate cell division after a threshold is reached. rtTA affects cell growth by sequestering molecules that would be used to drive the cell cycle forward. After division, molecules are reapportioned between the mother and daughter cell based on the binomial probability distribution. (B) Simulations of PF cells incorporating stochastic biochemical transitions and cell division. Simulations are "gated" so that cells with 60-70 cell counters are used to create histograms. Histograms are created from log-binning yEGFP::ZeoR protein concentrations, to make them consistent with flow cytometry measurements. (C) Time course tracking of live cell fluorescence by microscopy in a microfluidic chamber is consistent with a model where cells predominantly switch to the high expressor state, but predominantly reproduce in the low expressor state.*

## References


1. Becskei A, Seraphin B, Serrano L (2001) Positive feedback in eukaryotic gene networks: cell differentiation by graded to binary response conversion. Embo J 20: 2528-2535.
2. Becskei A, Kaufmann BB, van Oudenaarden A (2005) Contributions of low molecule number and chromosomal positioning to stochastic gene expression. NatGenet 37: 937-944.
3. Baron U, Gossen M, Bujard H (1997) Tetracycline-controlled transcription in eukaryotes: novel transactivators with graded transactivation potential. Nucleic Acids Res 25: 2723-2729.
4. Urlinger S, Baron U, Thellmann M, Hasan MT, Bujard H, et al. (2000) Exploring the sequence space for tetracycline-dependent transcriptional activators: novel mutations yield expanded range and sensitivity. ProcNatlAcadSciUSA 97: 7963-7968.
5. Garner DL, Gledhill BL, Pinkel D, Lake S, Stephenson D, et al. (1983) Quantification of the X- and Y-chromosome-bearing spermatozoa of domestic animals by flow cytometry. Biol Reprod 28: 312-321.
6. Kosugi Y, Ikebe J, Shitara N, Takakura K (1986) Graphical presentation of multidimensional flow histogram using hexagonal segmentation. Cytometry 7: 291-294.
7. Shapiro H (2003) Flow cytometry: problems, parameters, probes, and principles. Practical Flow Cytometry. 4th ed. pp. 18.
8. Murphy KF, Adams RM, Wang X, Balazsi G, Collins JJ (2010) Tuning and controlling gene expression noise in synthetic gene networks. Nucleic Acids Res 38: 2712-2726.
9. Nevozhay D, Adams RM, Murphy KF, Josic K, Balazsi G (2009) Negative autoregulation linearizes the dose-response and suppresses the heterogeneity of gene expression. Proc Natl Acad Sci U S A 106: 5123-5128.
10. Ferrell JE, Xiong W (2001) Bistability in cell signaling: How to make continuous processes discontinuous, and reversible processes irreversible. Chaos 11: 227-236.
11. Tan C, Marguet P, You L (2009) Emergent bistability by a growth-modulating positive feedback circuit. Nat Chem Biol 5: 842-848.
12. Ebeling W, Sokolov I (2005) Random Walk Approaches. Statistical Thermodynamics and Stochastic Theory of Nonequilibrium Systems. pp. 194-195.
13. Talkner P (1987) Mean first passage time and the lifetime of a metastable state. Zeitschrift für Physik B Condensed Matter 68: 201-207.
14. Gillespie DT (2000) The Chemical Langevin Equation. J Chem Phys 113: 297-306.
15. Ebeling W, Sokolov IM (2005) Statistical Thermodynamics and Stochastic Theory of Nonlinear Systems Far from Equilibrium: World Scientific.
16. Kramers HA (1940) Brownian motion in a field of force and the diffusion model of chemical reactions. Physica 7: 284-304.
17. Risken H, Frank T (1996) The Fokker-Planck Equation: Methods of Solutions and Applications: Springer.
18. Gilbert DM, Heery DM, Losson R, Chambon P, Lemoine Y (1993) Estradiol-inducible squelching and cell growth arrest by a chimeric VP16-estrogen receptor expressed in Saccharomyces cerevisiae: suppression by an allele of PDR1. Mol Cell Biol 13: 462-472.
19. Kudla G, Murray AW, Tollervey D, Plotkin JB (2009) Coding-sequence determinants of gene expression in Escherichia coli. Science 324: 255-258.

20. Scott M, Gunderson CW, Mateescu EM, Zhang Z, Hwa T (2010) Interdependence of cell growth and gene expression: origins and consequences. Science 330: 1099-1102.
21. Yeh PJ, Hegreness MJ, Aiden AP, Kishony R (2009) Drug interactions and the evolution of antibiotic resistance. Nat Rev Microbiol 7: 460-466.
22. Goldoni M, Johansson C (2007) A mathematical approach to study combined effects of toxicants in vitro: evaluation of the Bliss independence criterion and the Loewe additivity model. Toxicol In Vitro 21: 759-769.
23. Greco WR, Bravo G, Parsons JC (1995) The search for synergy: a critical review from a response surface perspective. Pharmacol Rev 47: 331-385.
24. Acar M, Becskei A, van Oudenaarden A (2005) Enhancement of cellular memory by reducing stochastic transitions. Nature 435: 228-232.
25. Acar M, Mettetal JT, van Oudenaarden A (2008) Stochastic switching as a survival strategy in fluctuating environments. Nat Genet 40: 471-475.
26. Novick A, Weiner M (1957) Enzyme induction as an all-or-none phenomenon. Proc Natl Acad Sci USA 43: 553-566.
27. Thattai M, van Oudenaarden A (2004) Stochastic gene expression in fluctuating environments. Genetics 167: 523-530.
28. Kussell E, Leibler S (2005) Phenotypic diversity, population growth, and information in fluctuating environments. Science 309: 2075-2078.
29. Kar S, Baumann WT, Paul MR, Tyson JJ (2009) Exploring the roles of noise in the eukaryotic cell cycle. Proc Natl Acad Sci U S A 106: 6471-6476.
30. Fantes PA (1977) Control of cell size and cycle time in Schizosaccharomyces pombe. J Cell Sci 24: 51-67.
31. Kamionka A, Bogdanska-Urbaniak J, Scholz O, Hillen W (2004) Two mutations in the tetracycline repressor change the inducer anhydrotetracycline to a corepressor. Nucleic Acids Res 32: 842-847.
32. To TL, Maheshri N (2010) Noise can induce bimodality in positive transcriptional feedback loops without bistability. Science 327: 1142-1145.
33. Ramsey S, Orrell D, Bolouri H (2005) Dizzy: stochastic simulation of large-scale genetic regulatory networks (supplementary material). J Bioinform Comput Biol 3: 437-454.
